# Cleavage of E-cadherin by porcine respiratory bacterial pathogens facilitates airway epithelial barrier disruption and bacterial paracellular transmigration

**DOI:** 10.1080/21505594.2021.1966996

**Published:** 2021-09-05

**Authors:** Qi Cao, Wenbin Wei, Huan Wang, Zesong Wang, Yujin Lv, Menghong Dai, Chen Tan, Huanchun Chen, Xiangru Wang

**Affiliations:** a State Key Laboratory of Agricultural Microbiology, College of Veterinary Medicine, Huazhong Agricultural University, Wuhan, Hubei, China; b Key Laboratory of Preventive Veterinary Medicine in Hubei Province, The Cooperative Innovation Center for Sustainable Pig Production, Wuhan, Hubei, China; c College of Veterinary Medicine, Henan University of Animal Husbandry and Economy, Zhengzhou, Henan, China; d Key Laboratory of Development of Veterinary Diagnostic Products, Ministry of Agriculture of the People’s Republic of China, Wuhan, Hubei, China; e International Research Center for Animal Disease, Ministry of Science and Technology of the People’s Republic of China, Wuhan, Hubei, China

**Keywords:** Airway epithelial barrier, porcine respiratory disease complex, paracellular transmigration, *Glaesserella parasuis*, HtrA, E-cadherin

## Abstract

Airway epithelial cells are the first line of defense against respiratory pathogens. Porcine bacterial pathogens, such as *Bordetella bronchiseptica, Actinobacillus pleuropneumoniae, Glaesserella* (*Haemophilus*) *parasuis*, and *Pasteurella multocida*, breach this barrier to lead to local or systematic infections. Here, we demonstrated that respiratory bacterial pathogen infection disrupted the airway epithelial intercellular junction protein, E-cadherin, thus contributing to impaired epithelial cell integrity. E-cadherin knocking-out in newborn pig tracheal cells via CRISPR/Cas9 editing technology confirmed that E-cadherin was sufficient to suppress the paracellular transmigration of these porcine respiratory bacterial pathogens, including *G. parasuis, A. pleuropneumoniae, P. multocida*, and *B. bronchiseptica*. The E-cadherin ectodomain cleavage by these pathogens was probably attributed to bacterial HtrA/DegQ protease, but not host HtrA1, MMP7 and ADAM10, and the prominent proteolytic activity was further confirmed by a serine-to-alanine substitution mutation in the active center of HtrA/DegQ protein. Moreover, deletion of the *htrA* gene in *G. parasuis* led to severe defects in E-cadherin ectodomain cleavage, cell adherence and paracellular transmigration *in vitro*, as well as bacterial breaking through the tracheal epithelial cells, systemic invasion and dissemination *in vivo*. This common pathogenic mechanism shared by other porcine respiratory bacterial pathogens explains how these bacterial pathogens destroy the airway epithelial cell barriers and proliferate in respiratory mucosal surface or other systemic tissues.

## Introduction

The mucosal barrier is composed of one or more layers of epithelial cells that have distinct apical and basolateral surfaces with specialized functions [1]. These cells form a permeability barrier between biological compartments that serve as both physical and immunological barriers to invading pathogens [2,3]. However, pathogens were able to circumvent the epithelial cell barrier by using diverse strategies, such as transcytosis, hijacking the epithelial cell biological process, or disrupting cell-to-cell junctional proteins [4–6]. Respiratory disease in pigs is arguably the most important health concern in pig industry nowadays which leads to significant economic losses [7]. Generally, viral and bacterial infections, as well as poor feeding managements, collectively result in porcine respiratory disease complex [7]. Among these bacterial pathogens, it is common to categorize them as either the primary bacterial pathogens, such as *A. pleuropneumoniae* and *B. bronchiseptica* which are capable of subverting respiratory defense mechanisms and establishing infection on their own, or the opportunistic bacterial pathogents, such as *G. parasuis* and *P. multocida* which take advantage of the virulence mechanisms of primary pathogens to establish infection [8]. Their successful colonizing of the upper respiratory tract or breaking through the airway epithelial barrier account for their local or systemic infections including septicemia, pleuropneumoniae, fibrinous polyserositis, meningitis, polyarthritis, pneumonia, and the death as well [7].

The tracheal epithelial cell plays important role in the defense of respiratory tract under multiple stimulations [9]. It provides a direct biological barrier against the invading microorganisms and inhaled particulates by virtue of the presence of intercellular junctions and mucociliary clearance [10]. Intercellular junctions, including tight junctions and adherens junctions, are crucial structures for the formation and maintenance of epithelial barrier functions to protect against extraneous pathogens [11]. Tight junctions sit below the apical membrane on lateral surfaces and some tight junction proteins are transmembrane proteins, including Occludin, Claudins, and junctional adhesion molecules, which are connected to zonula occludins family (ZO-1, ZO-2, and ZO-3) and finally to the actin backbone [12]. Adherens junctions are located below tight junctions and are involved in maintaining cell-cell communication, cell polarization, and the signaling transduction-involved transcriptional regulation [13,14]. At adherens junctions, E-cadherin is the key molecule that facilitates the establishment of epithelial barrier functions. It has five extracellular domains, a transmembrane domain, and an intracellular domain [15], in which the extracellular domains establish homophilic interactions in *cis* and *trans* that require calcium ion to the linker region between cadherin situated within two opposing cell membranes [16]. However, this homophilic interactions of E-cadherin between two adjacent cell membranes could be hijacked or disrupted by certain pathogens [1,17].

The serine protease HtrA has been identified as a novel secreted protein that plays important roles in the process of multiple pathogen infection. *Campylobacter jejuni* HtrA protein was shown important in the stress tolerance as well as host cell adhesion, invasion, and transmigration [18]. The HtrA secreted by *Helicobacter pylori* cleaved the cell-to-cell adherens junction protein E-cadherin and tight junction proteins Occludin and Claudin-8 [19], thereby allowing bacterial transmigration across the polarized epithelial cells and injecting virulence factors into host cells [6]. The HtrA of *Borrelia burgdorferi* not only decreased bacterial swarm motility and pyruvate production, but also degraded the host extracellular matrix (ECM) and stimulated the release of inflammatory cytokines in chondrocytes [20,21]. The protease HtrA in respiratory pathogen *G. parasuis* contributed to bacterial temperature stress tolerance, serum complement resistance, and virulence [22]. Recently, one study has indicated that *G. parasuis* infection activates the canonical Wnt/β-catenin signaling pathway leading to the disruption of epithelial barrier, with a sharp degradation of E-cadherin and an increase of the epithelial cell monolayer permeability [23]. However, the precise mechanism by which porcine respiratory bacterial pathogens destroy E-cadherin and cross the tracheal epithelial barrier is currently unclear.

In this work, we described the molecular process of the airway epithelial barrier disruption by porcine respiratory bacterial pathogens. We demonstrated the key contribution of the adherens junction protein E-cadherin to tracheal epithelial integrity, while bacterial secreted protease HtrA specifically cleaved intercellular E-cadherin and promoted bacterial paracellular transmigration. Characterization of this common pathogenic mechanism by porcine bacterial pathogens shall be beneficial for future prevention and control of these porcine respiratory infections.

## Materials and methods

### Cell culture

HEK293T cells were obtained from the American Type Culture Collection (Cat. number CRL-11,268) and grown in Dulbecco’s modified Eagle’s medium (DMEM, high-glucose) (Life Technologies Corporation, Gaithersburg, MD, USA) supplemented with 10% fetal bovine serum (GeminiBio, California, USA). NPTr cells [24] were grown in DMEM supplemented with 100 U/mL penicillin and 100 U/mL streptomycin (Solarbio, Beijing, China) containing 10% fetal bovine serum. Cells were cultured at 37°C with 5% CO_2_.

### Bacterial culture

*G. parasuis* (CF7066), *A. pleuropneumoniae* (S4074), and *B. bronchiseptica* (Bb1701079) were cultured at 37°C on tryptic soy agar (TSA) or in tryptic soy broth (TSB) (Difco Laboratories, Detroit, USA) containing 10 μg/mL NAD and 5% inactivated bovine serum (TSB/V/S or TSA/V/S). *P. multocida* (HB03) was grown on TSA or in TSB with 5% inactivated bovine serum at 37°C. Detailed bacterial strains and plasmids information are described in S1 Table.

### htrA *deletion mutant construction and growth analysis*

The CF7066*ΔhtrA* (*ΔhtrA*) mutant was generated via allelic exchange [25] by replacing the *htrA* gene with a kanamycin cassette (all primer sequences for *htrA* deletion are listed in Table S2). The growth of wild-type (WT) and *ΔhtrA* mutant was compared as described previously [22], with some modifications. Briefly, WT and *ΔhtrA* strains were grown in 5 mL of TSB/V/S and diluted in the same medium to an OD_600_ value of 1.0. The diluted cultures were then inoculated at 1:1000 ratio into 200 μl fresh TSB/V/S medium and the growth curve was conducted using a Bioscreen C apparatus (Oy Growth curves Ab Ltd, Helsinki, Finland).

### Bacterial infection

NPTr cells were seeded at 3.0 × 10^5^/well in 3.5 cm^2^ dishes containing DMEM without antibiotics. Before the infection experiments, the medium was replaced with fresh serum-free medium. When needed, Batimastat (20 μM, MedChemExpress, New Jersey, USA), TAPI-0 (25 μM, Sigma-Aldrich, St. Louis, MO, USA), or GI254023X (1 μM, MedChemExpress) were added 1 h before infection. For infection, serum-starved cells were infected at a multiplicity of infection (MOI) of 100 with *G. parasuis, P. multocida*, or *B. bronchiseptica*, and at an MOI of 1 with *A. pleuropneumoniae*. Cells were collected at specific time points for Western blot analysis.

### siRNA and transfection

NPTr cells were seeded into 6-well plates and cultured until reaching 60% confluence. Cells were transiently transfected with the corresponding siRNA by using the jetPrime® Transfection Reagent (Polyplus-transfection, Illkirch, France). After 24 h of transfection, the silencing efficiency was determined by Western blot. The specific siRNAs were used as follows: ADAM10 siRNA sense, 5′CCAGCAGAGAGAUAUAUUATT3′; antisense, 5′UAAUAUAUCUCUCUGCUGGTT3′; HtrA1-1 siRNA, sense, 5′CCAACAGUUUGCGC-CACAATT3′; antisense, 5′UUGUGGCGCAAACUGUUGGTT3′; HtrA1-2 siRNA, sense, 5′GCCAUCAUCAACUAUGGAATT3′; antisense, 5′UUCCAUAGUUGAUGAUGGCTT3′; HtrA1-3 siRNA, sense, 5′CCGUGAUUCCCGAAGAAAUTT3′; and antisense, 5′AUUUCUUCGGGAAUCACGGTT3′.

### Electric Cell-substrate Impedance Sensing (ECIS) assays

NPTr cells were seeded at a density of 1.0 × 10^5^/well on the collagen-coated, gold-plated electrodes with 8-well chamber sliders. The sliders were linked to the ECIS device (Applied BioPhysics, Troy, NY, USA) and were continuously measured to monitor the formation of epithelial barrier in a real-time manner. After maximal steady-state readings of transepithelial electronic resistance (TEER) had been reached, at which was indicative of maximal polarity and barrier function, *G. parasuis, A. pleuropneumoniae, P. multocida, B. bronchiseptica*, or the recombinant HtrA/DegQ protein were added to the ECIS chamber. The influence of these stimulations on the epithelial barrier was reflected by real-time monitoring of TEER values change and auto-recorded in the ECIS software.

### Bacterial transmigration assays

NPTr cells were cultured on 0.33 cm^2^ Transwell inserts (Corning, New York, USA) with 3 μm pore size at a density of 7.5 × 10^4^/well. The cells were grown to confluent monolayers and then incubated for another 5 d to allow cell polarization. The cells were infected in the apical compartment at an MOI of 2 with *G. parasuis, P. multocida*, or *B. bronchiseptica*, or at an MOI of 1 with *A. pleuropneumoniae* for 2 h and 5 h. After apical infection, the number of transmigrated bacteria was quantified in aliquots from the basal chambers and CFUs were counted on plates.

### Adhesion and invasion assays

NPTr cells were seeded at 1.0 × 10^5^/well in 24-well plates and incubated at 37°C under 5% CO_2_ as described previously [26,27], with some modifications. One hour before the infection, the cell culture medium was replaced by the serum-free media, and cells were then infected with *G. parasuis, P. multocida*, or *B. bronchiseptica* at an MOI of 100, or with *A. pleuropneumoniae* at an MOI of 1. The plates were centrifuged at 800 × *g* for 10 min to facilitate contact of bacteria to cell surface. After 1 h, 2 h, or 3 h post infection, cells were washed three times with 1 mL of phosphate-buffered saline (PBS) per well to remove non-adherent bacteria. To determine the total amount of adherent bacteria, the infected cells were lysed with 0.25% trypsin/0.02% EDTA and the CFU were enumerated by plating after serial dilutions. To determine the number of invasive bacteria, the infected monolayers were moreover incubated with 1 mL of DMEM medium containing 5 μg/mL gentamicin and 100 μg/mL penicillin G for 30 min to kill extracellular surface-attached bacteria. Adhesion was defined as the total number of the cell-associated bacteria and was expressed as the relative percentage of their input. Invasion was defined as the total number of intracellular bacteria and expressed as the relative percentage of the total inoculated bacteria.

### Cloning, mutagenesis, protein purification, and antibody production

*E. coli* BL21 (*DE3*) was transformed with a pET-28a plasmid expressing HtrA of *G. parasuis* (*Gp*HtrA, aa 29–459), DegQ of *A. pleuropneumoniae* (*Ap*DegQ, aa 28–464), or DegQ of *P. multocida* (*Pm*DegQ, aa 27–459) without signal peptide. Primer sequences used for the cloning were listed in Table 1. To generate inactive HtrA or DegQ protease (*Gp*HtrA S219A, *Ap*DegQ S221A, *Pm*DegQ S219A), site-directed mutagenic PCR was performed with serine to alanine mutation (HtrA^SA^/DegQ^SA^) in the active center as described previously [28] and verified by sequencing. Bacterial cells were cultured in 200 mL Luria broth (LB) supplemented with 50 μg/mL kanamycin at 37°C until the OD_600_ reached 0.6, and the isopropyl β-D-1-thiogalactopyranoside (IPTG) was added at a final concentration of 0.8 mM to induce the protein expression. Bacterial culture was pelleted at 6,000 × *g* for 15 min at 4°C and lysed in 20 mL PBS by sonication. The lysate was separated by centrifugation at 12,000 × *g* for 10 min. Recombinant proteins were purified from the supernatant by metal affinity chromatography using Ni Sepharose^TM^ 6 Fast Flow (GE Healthcare Life Sciences, Chicago, USA). Elution was carried out using imidazole buffer (50 mM Na_3_PO_4_ · 12H_2_O, 500 mM NaCl, and 500 mM imidazole, pH 7.4). A polyclonal antibody recognizing *Gp*HtrA was produced by immunization of mice with the recombinant *Gp*HtrA^WT^ protein.

**Table 1.** Proteins analyzed in this study

Proteins	Primer sequences (5′ to 3′) ^a^	Mutagenesis primer (5′ to 3′) ^b^
*Gp*HtrA	GGAATTCACATTGCCTACTGCTGTAAACG	CGTGGTAACGCCGGCGGCCCATTAA
	CCCAAGCTTATTAATGATTACATAGAAAT	TTAATGGGCCGCCGGCGTTACCACG
*Ap*DegQ	CTAGCTAGCACTTTACCGATTGCCATTGA	TAACGCGGGCGGTCCGTTA
	CCCAAGCTTTTGAAGAATTAAGTAAAAGTTTGC	CCGCCCGCGTTACCTTGG
*Pm*DegQ	CTAGCTAGCTCCTTACCAACTCATGTTGA	GGAAATGCCGGAGGTCCTTT
	CGGGATCCGCTTGAATAAGAAGATAGAAATTCG	ACCTCCGGCATTTCCGCG
ΔN-HtrA	GGAATTCGGTTCTGGTGTAATTATTGAT	
	CCCAAGCTTATTAATGATTACATAGAAAT	
E-Cad-NTF	CCCAAGCTTATGCAAGAGCCGGAGCCCTGC	
	CGGGATCCGCATACGTGCACATCTAAGGTGGT	

^a^restriction recognition sites are underlined; ^b^ substituted nucleotides are underlined.

### In vitro *and* in situ *cleavage assays*

HEK293 cells were induced to produce the recombinant extracellular domain of porcine E-cadherin protein (rE-Cad-NTF-FLAG). The protein was then treated with equal volume of 0.1 M glycine pH 3.0 for 10 min at room temperature followed by the addition of 1 M Tris-HCl pH 8.0, as described previously [29]. For *in vitro* cleavage assays, 100 ng of recombinant extracellular E-cadherin was incubated with 200 ng of recombinant HtrA/DegQ in 50 mM HEPES (pH 7.4) containing 1 mM EDTA at 37°C for 16 h. For *in situ* cleavage assays, 5 ng/μl or 50 ng/μl of recombinant HtrA^WT^/HtrA^SA^ or 50 ng/μl of recombinant DegQ^WT^/DegQ^SA^ were added to the cell culture for 24 h.

### Casein zymography

Bacterial lysates or recombinant HtrA/DegQ were loaded on 8% SDS-PAGE gels containing 0.1% casein and separated by electrophoresis under denaturing conditions. After protein separation, the gel was renatured in 2.5% Triton X-100 solution at room temperature for 1 h with gentle agitation, equilibrated in developing buffer (50 mM Tris-HCl, pH 7.4; 200 mM NaCl, 5 mM CaCl_2_, 0.02% Brij 35) at room temperature for 1 h with gentle agitation, and the gel was incubated overnight at 37°C in fresh developing buffer. Finally, transparent bands having caseinolytic activity were visualized by staining with 0.5% Coomassie Blue R250, as previously described [30].

### Sequence analysis

Protein sequences from *G. parasuis* HtrA (B8F6T4), *A. pleuropneumoniae* DegQ (A0A223MC71), *P. multocida* DegQ (A0A1E3XJJ4), and *B. bronchiseptica* DegQ (A0A2J9U7C5) were retrieved from the National Center for Biotechnology Information. Protein domain prediction was performed using SignalP ver5.0 and the Simple Modular Architecture Research Tool [31,32].

### Mass-spectrometry

Proteins in the negatively stained bands of *G. parasuis* were excised from gels and digested with trypsin (Promega, Madison, WI, USA). The resulting peptides were separated by Easy-nLC1000 (Thermo Fisher, Waltham, MA, USA). The obtained mass data were analyzed with Proteome Discoverer 1.4, using the UniProt database. Peptide tolerance was set to 50 ppm, and a maximum of two missed cleavage sites was allowed.

### E-cadherin knockout

The E-cadherin gene was knocked out in NPTr cells using CRISPR/Cas9 editing technology, as described previously [33]. For designing short guide RNA (sgRNA) pairs targeting the E-cadherin gene, the online CRISPR/Cas9 design tool (https://crispr.cos.uni-heidelberg.de/) was used for optimal sgRNA analysis. First, a 250 bp genome fragment from the early exons of target region was fed into the design tool. After computational analysis, suitable targets were identified and listed by ranking and scores, according to the prediction of their off-target potential. The NPTr cells were transfected with CRISPR/Cas9 plasmids (purchased from Nanjing YSY Biotech Co. LTD, China) containing E-cadherin sgRNA. The following sgRNA primers were used: sgRNA1 primers, sense, 5′CACCGCGTCGGTGCCCACTTTGAAT3′; antisense, 5′AAACATTCAAAGTGGGCACCGACGC3′; sgRNA2 primers, sense, 5′CACCGCCTTGGTGGACAGCTTCCTG3′; antisense, 5′AAACCAGGAAGCTGTCCACCAAGGC3′. The following sequencing primers (Identify-E-cadherin-F/R) were used to identify the E-cadherin knockout region, sense, 5′TCTTTGCTCTTCTGCCACCAGATTA3′, and the antisense, 5′CCAGGGATGCAGCCAAACATC3′.

### Detection of HtrA secretion

HtrA secretion assays were performed as described previously [34,35]. *G. parasuis* strain was suspended in TSB/V/S medium. To allow *Gp*HtrA secretion in the culture supernatant, the bacteria were incubated for 24 h with gentle agitation. At the indicated time points, the cellular and secreted proteins were separated by centrifugation at 1,500 × *g* for 10 min. The supernatants were again centrifuged for 15 min at 13,000 × *g* to remove cell debris and then were sterile-filtered. For *Gp*HtrA secretion assays in the presence of NPTr cells, cells were cultured in 3.5 cm^2^ dishes and *G. parasuis* suspension was added to each dish. At different time points, the supernatant of cell cultures were harvested and immediately centrifuged (13,000 × *g* for 10 min at 4°C) to collect both the supernatant and bacterial cell pellet.

### Western blot

Cells were collected and lysed using radioimmunoprecipitation assay lysis buffer containing phenylmethylsulfonyl fluoride (Beyotime, Shanghai, China). Cell samples were centrifuged for 10 min at 13,000 × *g* at 4°C to separate the pellets, and the supernatants were analyzed using Western blot. For bacterial cells, the bacteria were harvested in sterile PBS supplemented with 0.1% Triton X-100 and sonicated to prepare bacterial lysates. Bacterial fractions or infected cells were mixed with 5× SDS-PAGE buffer and boiled for 10 min. Proteins were separated by SDS-PAGE on 12% polyacrylamide gels and electrotransferred to polyvinylidene difluoride membranes (Immobilon-P Millipore) using standard procedures (Bio-Rad, Hercules, CA, USA). The membranes were blocked with 5% bovine serum albumin (BSA, Sigma-Aldrich) for 2 h and incubated overnight with primary antibodies at 4°C. Subsequently, the membranes were washed with Tris-buffered saline-Tween 20 and incubated with the corresponding secondary antibody for 1 h. Finally, the membranes were visualized using the ChemiDoc XRS system (Bio-Rad).

### Antibodies

For Western blot or immunofluorescence analysis, the following antibodies were used: anti-ADAM10 (A10438; ABclonal, Wuhan, China), anti-E-cadherin full length (3195; Cell Signaling Technology, Danvers, MA, USA), anti-E-cadherin NTF (A3044, ABclonal), anti-claudin-1 (ab129119; Abcam, Cambridge, MA, USA), anti-occludin (13,409-1-AP; Proteintech, Wuhan, China), anti-ZO-1 (21,773-1-AP; Proteintech), anti-HtrA1 (ab199529; Abcam), anti-MMP7 (10,374-2-AP; Proteintech), anti-FLAG (20,543-1-AP; Proteintech), and anti-GAPDH (10,494-1-AP; Proteintech). Horseradish peroxidase (HRP)-labeled goat anti-rabbit (bs-0295 G-HRP; Bioss, Beijing, China), HRP-labeled goat anti-mouse (bs-0296 G-HRP; Bioss), and Cy3-conjugated goat anti-rabbit (A0516; Beyotime) secondary antibodies were used.

### Animal experiments

Eleven 25-day-old piglets were allocated randomly to WT, *ΔhtrA* and PBS groups. Each group contained four piglets and the remaining three were allocated to the PBS control group. The piglets were intranasally inoculated with 2 mL (1 mL per nostril) sterile PBS or with 2.0 × 10^10^ CFU of WT or *ΔhtrA* strains (2 mL), respectively. At 1, 3, and 5 days post infection, one or two piglets from each group was euthanized, and swabs from nasal cavities, tracheal, lungs, blood, and joints were collected and subjected to bacterial isolation. After 36 h of incubation at 37°C, *G. parasuis* growth was semiquantified by assigning a score of 3 to samples with confluent growth, 2 to samples yielding colonies from 20 to 200, 1 to samples yielding 1 to 19 colonies, and 0 to samples without bacterial growth [36].

### Immunofluorescence and HE staining

Trachea and lung samples were fixed by 20% buffered neutral formalin and subsequently embedded in paraffin. Paraffin-embedded tissues were sectioned at 3 μm and stained with HE. For the tissue immunofluorescence assay, the sections were blocked with 5% goat serum in PBS and then incubated with the corresponding primary antibody (1:200 dillution) for overnight at 4°C. After wash, the sections were incubated with Cy3-labeled goat anti-rabbit IgG (1:200 dilution) for 1 h at 37°C. The 4,6-Diamindino-2-phenylindole (DAPI) was used to stain cell nucleus. Finally, the cells were visualized under an Olympus confocal fluorescence microscope (Tokyo, Japan). For the cell immunofluorescence assay, NPTr cells were cultured on coverslips, fixed (4% paraformaldehyde), and blocked with PBS containing 5% BSA for 2 h at 37°C. The cells were then incubated with the corresponding primary antibody overnight at 4°C. After five washes with PBS, the secondary antibody was used for 1 h, and then the slides were mounted in ProLong antifade reagent (Beyotime). Samples were analyzed using confocal laser scanning microscopy with a Zeiss LSM 880 Meta confocal microscope (Carl Zeiss, Jena, Germany).

### Immunohistochemistry (IHC)

Formalin-fixed, paraffin-embedded trachea and lung tissues were cut into sections of 3 μm. For antigen retrieval, the tissues was performed with 10 mM citrate buffer at 98°C for 20 min. After blocking with 3% BSA in PBS for 30 min at 37°C, *G. parasuis* detection was performed with the primary antibody reactions using anti-*G. parasuis* antibody (1:200 dillution), which was obtained in rabbit by hyper-immunization with methanal-killed *G. parasuis* strain CF7066. After overnight incubation at 4°C, the sections were washed and incubated with an HRP-labeled goat anti-rabbit IgG (1:200 dilution) for 1 h at 37°C. After washing, slides were revealed with a solution of 0.05% diaminobenzidine tetrahydrochloride (DAB). Then, tissue sections were counterstained with hematoxylin and mounted with distrene-plasticizer-xylene (DPX). The images were captured by fluorescence microscope (EVOS FL ATUO, Life Technology).

### RNA extraction and quantitative real-time PCR analysis

Total cellular or bacterial RNAs were extracted by using the TRIzol reagent (Invitrogen, Carlsbad, CA, USA), and cDNA was prepared using the PrimeScript^TM^ RT reagent kit with gDNA Eraser (Takara Bio Inc., Shiga, Japan). Real-time PCR was performed with a qTOWER^3^/G quantitative real-time PCR thermal cycler (Analytik Jena, Jena, Germany) by using Power SYBR Green PCR master mix (Applied Biosystems, CA, USA), according to the manufacturer’s instructions. The following quantitative real-time PCR primers were used: E-cadherin primers, sense, 5′GAGGGATGCTGCCAACTG3′; antisense, 5′GCCGTGTATGTGCTGTTCTT3′; GAPDH primers, sense, 5′CACAGTCAAGGCGGAGAAC3′; antisense, 5′CGTAGCACCAGCATCACC3′; *htrA* primers, sense, 5′GGCATTAGGTCGTTCAAC3′; antisense, 5′CTTCACTTCTCCAAACTCAAT3′; 16S rRNA primers, sense, 5′TGAAGTCGGAATCGCTAGTA3′; and antisense, 5′CCTACGGTTACCTTGTTACG3′.

### Statistical analysis

All data were collected from at least three independent experiments in triplicate. Data were combined and represented as the mean ± SD. The results were analyzed by various statistical tests using GraphPad Prism version 7. Differences were considered significant when *P* is < 0.05, * indicates *P* < 0.05, ** indicates *P* < 0.01, and *** indicates *P* < 0.001.

## Results

### *Porcine respiratory bacterial pathogen infection disrupted the integrity of airway epithelial barrier* in vivo *and* in vitro

**Figure 1.**
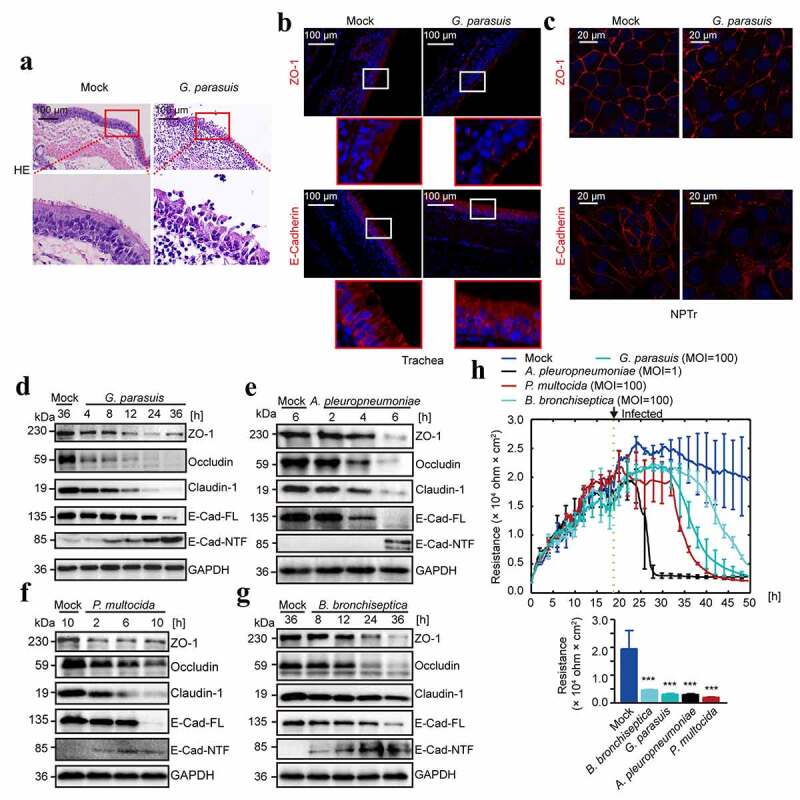
Porcine respiratory bacterial infection disrupted the integrity of respiratory epithelial barrier *in vivo* and *in vitro.*

The influence of porcine respiratory bacterial pathogens on the tracheal tissue of piglets was first investigated. Piglets were intranasally challenged with *G. parasuis* virulent strain CF7066 as a representative. As the Hematoxylin and Eosin (HE) staining shown, *G. parasuis* infection caused the damage of tracheal epithelium, demonstrated by the loss of respiratory tract cilia and disruption of tracheal epithelial arrangement, while in contrast the control group displayed an intact tracheal epithelial layer and normal tissue architecture (Figure 1(a)). By immunofluorescence to visualize these tracheal epithelial cell-to-cell junctions, we observed that *G. parasuis* largely destroyed the expression and distribution of the epithelial tight junction protein ZO-1 and the adherens junction protein E-cadherin in the pig tracheal tissues as well as the newborn pig tracheal (NPTr) cell line (Figure 1(b,c)). We next evaluated the time-dependent expression of these tight junction and adherens junction proteins in NPTr cells during the *in vitro* infection, and found the obvious decrease of these junction-associated proteins, including ZO-1, Occludin, Claudin-1, and E-cadherin, along with the infection (Figure 1(d)), and all these porcine respiratory bacterial pathogens including *G. parasuis, A. pleuropneumoniae, P. multocida*, and *B. bronchiseptica* exhibited the similar influence at the indicated time points (Figure 1(e,g); see also Figure S1). Noticeably, The ectodomain shedding of E-cadherin was meanwhile observed accompany with the decrease of its full-length E-cadherin (E-Cad-FL) in whole cell lysates, as demonstrated by the increasing extracellular N-terminal fragment (NTF) in cell supernatant (Figure 1(d,g)). Since both tight junctions and adherens junctions play crucial roles in the maintenance of epithelial cell barrier function [12]. We additionally checked the transepithelial electrical resistance (TEER) of the NPTr monolayer cells in response to these porcine respiratory bacterial pathogens by electric cell-substrate impedance sensing (ECIS) system. As demonstrated, the TEER values of monolayer NPTr cells infected with these bacteria exhibited the significant decreased resistance compared with the unchallenged cells during the observation (Figure 1(h)), suggesting the infection-caused damage to this epithelial cells barrier. These evidences collectively confirmed that porcine respiratory bacterial pathogens could efficiently disrupt the cell-to-cell junctions and impair the integrity of tracheal epithelial cells barrier.

(a) HE staining of the tracheal epithelium in response to *G. parasuis* infection. Scale bar indicated 100 μm. (b) ZO-1 (red) and E-cadherin (red) staining in trachea of pigs with or without infection. Scale bar indicated 100 μm. (c) ZO-1 and E-cadherin staining in NPTr cells in response to 24 h of infection. Scale bar indicated 20 μm. (d-g) The cleavage or degradation of junction-associated proteins by porcine respiratory bacterial pathogens including *G. parasuis* CF7066 strain (MOI = 100, in response to 36 h of infection), *A. pleuropneumoniae* S4074 strain (MOI = 1, in response to 6 h of infection), *P. multocida* HB03 strain (MOI = 100, in response to 10 h of infection), and *B. bronchiseptica* Bb1701079 strain (MOI = 100, in response to 36 h of infection). GAPDH in whole cell lysates was detected as the loading control. (h) Real-time measurement of the transepithelial electrical resistance (TEER) in the monolayer NPTr cells in response to these porcine respiratory bacterial pathogens, respectively. The arrow indicated the inoculation of these bacterial pathogens (at 18 h). The TEER values (Ohm × cm^2^) at each point represented the average values from three independent experiments, and each independent experiment includes triplicate wells. Endpoints of the experiments data were shown as mean ± SD. Statistical significance was analyzed using the one-way ANOVA (Dennett’s multiple comparisons test, *** *p* < 0.001).

### E-cadherin was essential in porcine respiratory bacterial transmigration of the tracheal epithelial cells

The adherens junction protein E-cadherin plays multiple roles in defense against bacterial invasion, paracellular transmigration, and maintenance of epithelial barrier [17,37]. Since we observed the significant decrease of E-cadherin as well as its ectodomain shedding upon the infection, we next investigated the influence of E-cadherin in these porcine respiratory bacterial infection of tracheal epithelial cells. CRISPR/Cas9 technology was applied to specificly knockout E-cadherin in NPTr cells by targeting exon 3 (Figure S2A and S2B) [38]. The expression of E-cadherin in each single cell clone was verified using Western blot with a full-length E-cadherin specific antibody (Figure S2C), and the selected clone 2 C9 was further tested for the stability of E-cadherin knockout (E-Cad KO) after several passages (Figure S2D).

**Figure 2.**
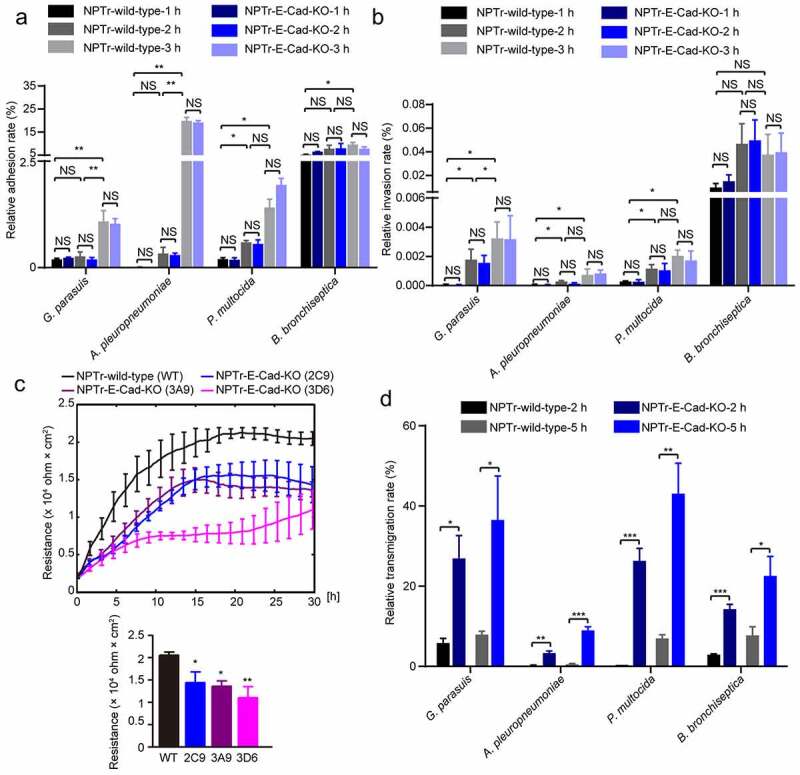
E-cadherin played key roles in preventing porcine respiratory bacterial transmigration of NPTr cells

Subsequently, the adhesion and invasion of both wild-type and E-Cad KO NPTr cells by these porcine respiratory bacterial pathogens were compared along with the infection. As shown, the bacterial adhesion and invasion of E-Cad KO cells at different time points were nearly the same level as that of the wild-type cells, suggesting that the deletion of E-cadherin did not influence the bacterial adhesion and invasion of NPTr cells (Figure 2(a,b)). Moreover, the effects of E-cadherin deletion on monolayer TEER were further evaluated via ECIS, and the significant lower TEER values in different E-Cad KO constructs were detected during the observation, compared to the wild-type cells (Figure 2(c)). As expected, by using Transwell chambers, the bacterial transmigration of E-Cad KO cells were significantly higher than that of the wild-type cells, at different time points (Figure 2(d)). These data supported the important role of E-cadherin in paracellular transmigration by these respiratory bacterial pathogens.

(a and b) The adhesion and invasion of both wild-type and E-Cad KO NPTr cells by *G. parasuis* (MOI = 100), *P. multocida* (MOI = 100), *B. bronchiseptica* (MOI = 100), or *A. pleuropneumoniae* (MOI = 1) for 1 h, 2 h, or 3 h. Data were shown as mean ± SD from three independent experiments. Statistical significance was analyzed by using the two-way analysis of variance (ANOVA, Sidak’s multiple comparisons test, * *p* < 0.05, ** *p* < 0.01, NS, not significant). (c) Real-time measurement of the transepithelial electrical resistance (TEER) in wild-type cells as well as E-Cad KO cells (clones 2 C9, 3A9, and 3D6). The TEER values (Ohm × cm^2^) at each point represented the average values from three independent experiments, and each independent experiment includes triplicate wells. Endpoints of the experiments data were shown as mean ± SD. Statistical significance was analyzed using the one-way ANOVA (Dennett’s multiple comparisons test, * *p* < 0.05, ** *p* < 0.01). (d) The transmigration of both wild-type cells and E-Cad KO cells by *G. parasuis* (MOI = 2), *P. multocida* (MOI = 2), *B. bronchiseptica* (MOI = 2), or *A. pleuropneumoniae* (MOI = 1) at 2 h and 5 h. Data were shown as mean ± SD from three independent experiments. Statistical significance was analyzed by using the two-way ANOVA (Sidak’s multiple comparisons test, * *p* < 0.05, ** *p* < 0.01, and *** *p* < 0.001).

### Degradation of E-cadherin upon porcine respiratory bacterial pathogen infection was independent of the matrix metalloprotease 7 and a disintegrin and metalloprotease 10

**Figure 3.**
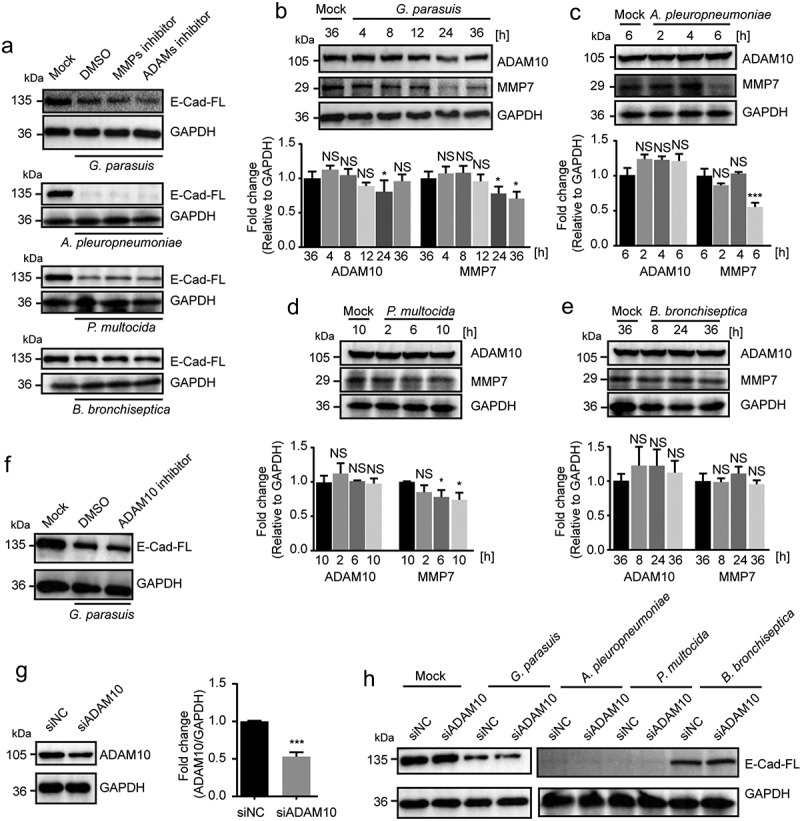
E-cadherin ectodomain shedding was independent of host proteases matrix metalloprotease 7 (MMP7) and a disintegrin and metalloprotease 10 (ADAM10) during porcine respiratory bacterial infection

Previous studies have demonstrated that ectodomain shedding of E-cadherin is assocated with the epithelial-to-mesenchymal transition in tumors and the inflammatory responses [39,40], and these effects are attributed to cellular matrix metalloprotease 7 (MMP7) and a disintegrin and metalloprotease 10 (ADAM10) [41–43]. We next wondered whether MMPs or ADAMs were involved in respiratory bacterial pathogen infection-caused E-cadherin degradation and ectodomain shedding. By using the MMPs inhibitor (20 μM, Batimastat) and ADAMs inhibitor (25 μM, TAPI-0), we observed both inhibitors did not alleviate the infection-caused E-cadherin degradation (Figure 3(a)). Moreover, we found that the MMPs member MMP7 was downregulated in response to the infection (except *B. bronchiseptica*), while the ADAM10 was almost not affected (Figure 3(b,e)). We further applied the ADAM10 selective inhibitor GI254023X (1 μM) as well as specific small interfering RNAs (siRNA) to inhibit its function (Figure 3(f,g)), and found that ADAM10 inhibition and knockdown could not significantly restore the E-cadherin degradation by these respiratory pathogens (Figure 3(f,h)). These findings excluded the involvement of host cell metalloproteases in E-cadherin degradation, and suggested the possible contribution of bacterial-derived protease to the infection-induced E-cadherin decrease as well as ectodomain shedding.

(a) The effects of MMPs selective inhibitor Batimastat (20 μM) and ADAMs family inhibitor TAPI-0 (25 μΜ) on the bacterial infection-induced ectodomain shedding of E-cadherin. *G. parasuis* and *B. bronchiseptica* infections were applied at an MOI of 100 for 24 h, *P. multocida* infection was applied at an MOI of 100 for 10 h, and *A. pleuropneumoniae* infection was applied at an MOI of 1 for 6 h. (b-e) Western blot determining the expression levels of ADAM10 and MMP7 in NPTr cells along with these porcine respiratory bacterial pathogens infection as above mentioned MOIs. MMP7 or ADAM10 bands densitometry were normalized to GAPDH and shown as mean ± SD from three independent experiments. Statistical significances were analyzed using the one-way ANOVA (Dennett’s multiple comparisons test, * *p* < 0.05, *** *p* < 0.001, NS, not significant). (f) The effect of ADAM10 specific inhibitor GI254023X (1 μM) on the *G. parasuis*-induced ectodomain shedding of E-cadherin. (g) Western blot demonstrating the siRNA knocking-down of ADAM10 in NPTr cells. Band densitometry was normalized to GAPDH and shown as mean ± SD from three independent experiments. Statistical significance was analyzed using Student’s *t* test (*** *p* < 0.001). (h) Western blot showed that ADAM10 siRNA interfering did not influence the ectodomain shedding of E-cadherin induced by *G. parasuis* and *B. bronchiseptica* (MOI of 100 for 24 h), *P. multocida* (MOI of 100 for 10 h), and *A. pleuropneumoniae* (MOI of 1 for 6 h). GAPDH was used as the loading control for the above blots.

### Bacterial protease HtrA/DegQ, but not host protease HtrA1, was responsible for E-cadherin ectodomain shedding in porcine respiratory bacterial infections

**Figure 4.**
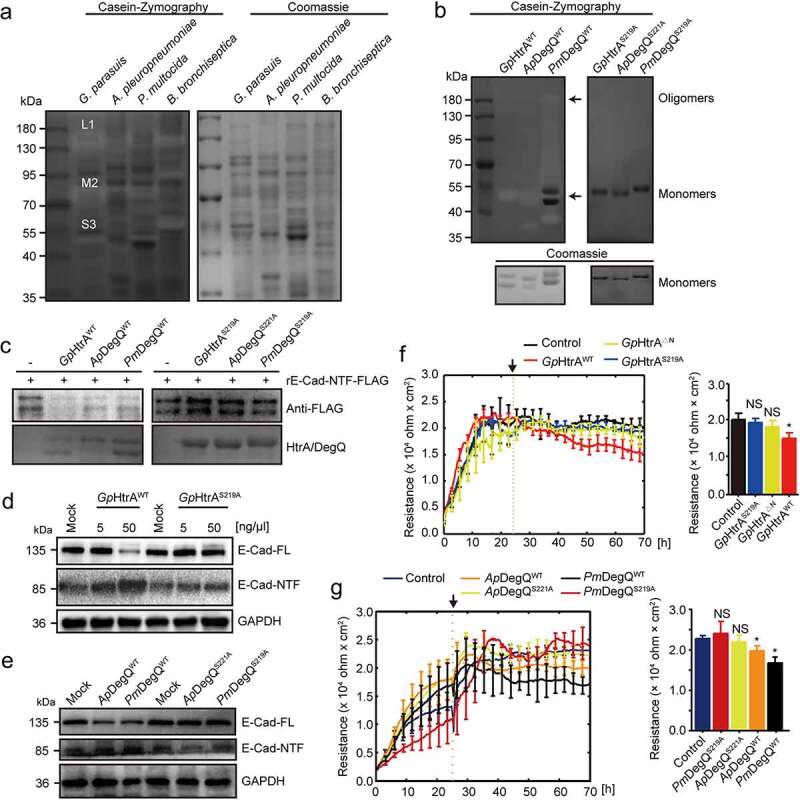
Bacterial HtrA/DegQ protein cleaved E-cadherin

We next sough to evidence if bacterial-derived protease mediated the degradation of E-cadherin. Whole bacterial lysates from these respiratory bacteria were analyzed in the SDS-PAGE gel containing casein under the non-reducing conditions. After renaturation and Coomassie Blue R250 staining, some transparent bands were appeared which indicating the protease activity (Figure 4(a)). These negatively stained proteolytic bands from *G. parasuis* sample were excised and analyzed by mass spectrometry, and the serine protease HtrA was identified as a possible contributor (Table S3). Noticeably, this protease HtrA was commonly found present in the other porcine respiratory bacteria *G. parasuis, A. pleuropneumoniae, P. multocida*, and *B. bronchiseptica*, and their amino acid sequences were highly conserved. *G. parasuis* HtrA showed 76%, 74%, and 39% identities with the DegQ from *A. pleuropneumoniae, P. multocida*, and *B. bronchiseptica* (Figure S3A).These sequences comprises an N-terminal signal peptide (SP) followed by a conserved trypsin-like peptidase domain including a catalytic triad composed of histidine, aspartate, and serine residues, and two Postsynaptic density of 95 kDa, Discs large, and Zonula occludens (PDZ) domains (PDZ1 and PDZ2) that were involved in substrate recognition, binding, and protein oligomerization (Figure S3B) [44]. We further evidenced that this HtrA protease could be secreted, by the demonstrations of a time-dependent increase in both the bacterial cultural supernatant and the cell supernatant after bacterial incubation (Figure S4A-S4C). Moreover, the recombinant wild-type HtrA protein of *G. parasuis*, the homolog DegQ of *A. pleuropneumoniae* and *P. multocida*, as well as their respective inactived mutant with a serine to alanine substitute (*Gp*HtrA^S219A^/*Ap*DegQ^S221A^/*Pm*DegQ^S219A^) in the active center were constructed and purified, and tested for their proteolytic activities using casein zymography. All the recombinant HtrA^WT^/DegQ^WT^ proteins exhibited caseinolytic activities, either as a monomer and/or as an oligomer. In contrast, their inactive mutants HtrA^SA^/DegQ^SA^ did not show any caseinolytic activities (Figure 4(b)). Importantly, the cleavage of these HtrA/DegQ proteins on E-cadherin-NTF were determined and found that HtrA^WT^/DegQ^WT^ efficiently cleaved the recombinant E-cadherin, as reflected by the significant reduction of the E-cadherin-NTF, but this E-cadherin-NTF cleavage was not observed by the inactive HtrA^SA^/DegQ^SA^ proteins (Figure 4(c)). The similar results were also observed in the E-cadherin cleavage *in situ*, that the HtrA^WT^/DegQ^WT^ protein mediated E-cadherin decrease and ectodomain release from cultured NPTr cells, while their HtrA^SA^/DegQ^SA^ proteins did not (Figure 4(d,e)). Besides, we checked the potential influence of the wild-type and mutant HtrA/DegQ proteins treatment on the monolayer NPTr cells by ECIS assay, and showed that the bacterial wild-type HtrA/DegQ obviously decreased the epithelial cells resistance while the *Gp*HtrA^S219A^ proteins, including HtrA with N-terminal (amino acid position at 29–95) deletion (*Gp*HtrA^ΔN^), *Ap*DegQ^S221A^ and *Pm*DegQ^S219A^, were unable to attenuate the epithelial cells resistance (Figure 4(f,g)).

(a) The protease activities of the lysates from *G. parasuis, A. pleuropneumoniae, P. multocida*, and *B. bronchiseptica* tested *via* casein zymography. Coomassie blue R250-stained SDS-PAGE gel was displayed as the loading. Negatively-stained protease bands L1, M2 and S3 were further analyzed by mass-spectrometry. (b) Active (WT) and inactive (Serine to Alanine mutation) recombinant HtrA/DegQ proteins were analyzed by casein zymography. Black arrows indicated the oligomers or monomers of HtrA^WT^/DegQ^WT^. (c) The *in vitro* cleavage of recombinant extracellular domains of porcine E-cadherin (rE-Cad-NTF-FLAG) by the active or inactive HtrA/DegQ proteins. E-cadherin shedding was shown as the decrease of recombinant E-cadherin by using anti-FLAG antibody. The HtrA/DegQ proteins were demonstrated by Coomassie Blue R250 staining. (d and e) Ectodomain shedding of E-cadherin from NPTr cells incubated with the active or inactive HtrA/DegQ proteins. The full-length E-cadherin (E-Cad-FL) was detected from cell lysates and the E-Cad-NTF was detected from the culture supernatant. GAPDH in whole cell lysates was shown as the loading control. (f and g) ECIS real-time monitoring the TEER values of the monolayer NPTr cells incubated with the active or inactive *Gp*HtrA (F), *Ap*DegQ or *Pm*DegQ (g). Black arrows indicated the addition of the HtrA/DegQ proteins (at 24 h). The TEER values (Ohm × cm^2^) at each point represented the average values from three independent experiments, and each independent experiment includes triplicate wells. Endpoints of the experiments data were shown as mean ± SD. Statistical significance was analyzed using the one-way ANOVA (Dennett’s multiple comparisons test, * *p* < 0.05, NS, not significant).

HtrA protein is widely distributed in mammalian and prokaryotic species, indicating that they might have functional similarities [45,46]. We therefore analyzed the possible involvement of host HtrA protein in this process. As found, the host cell HtrA protein, HtrA1, was not affected during the course of *G. parasuis* infection (Figure S4D). We additionally knocked down this host HtrA1 protein by siRNA approach (Figure S4E), and observed that the HtrA1 interfering or not did not influence the E-cadherin expression, no matter with or without bacterial infection (Figure S4F). These data confirmed that the bacterial protease HtrA/DegQ, but not porcine HtrA1 protease, acted specifically for E-cadherin ectodomain shedding in porcine respiratory bacterial infections.

### HtrA deletion significantly affected bacterial degradation of E-cadherin and bacterial transmigration

**Figure 5.**
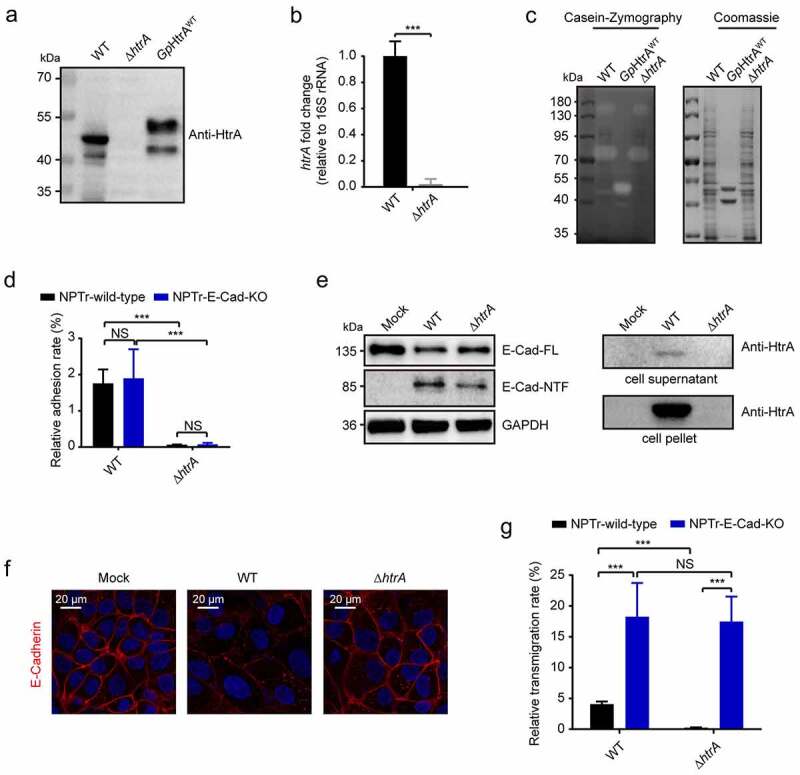
HtrA-mediated E-Cadherin cleavage was important for *G. parasuis* infection

Next, *G. parasuis htrA* deletion mutant was generated by using natural transformation system (Figure S5A). Western blot and qPCR assays were used to confirm the gene deletion in *ΔhtrA* strain (Figure 5(a,b)), which kept the similar growth rate as its WT (wild-type) strain (Figure S5B). Meanwhile, casein zymography experiment further supported the partial functional loss of the proteolytic activity in *ΔhtrA* strain (Figure 5(c)). With this *ΔhtrA* mutant strain, we subsequently found that the *ΔhtrA* strain exhibited a severe defect in the adhesion of both the wild-type NPTr cells and the E-Cad KO cells (Figure 5(d)). The *ΔhtrA* strain also exhibited an attenuated proteolytic effect on E-cadherin compared to the WT strain, as demonstrated by Western blot as well as immunofluorescence (Figure 5(e,f)). Accordingly, the *ΔhtrA* strain transmigration of the wild-type NTPr cells was significantly lower than the *G. parasuis* WT strain, despite no significance was observed on E-Cad KO cells between the WT strain and the *ΔhtrA* mutant (Figure 5(g)). These data further evidenced that HtrA acted essentially to facilitate bacterial cleavage of E-cadherin and the paracellular transmigration.

(a) The lysates of *G. parasuis* WT and *ΔhtrA* strains were validated for the expression of HtrA protein by Western blot with anti-HtrA antibody. The purified *Gp*HtrA^WT^ protein from *G. parasuis* was used as the control. (b) The transcriptional level of *htrA* gene in both WT and *ΔhtrA* strains, as detected by qPCR. Bacterial 16S rRNA was the reference control. Data were shown as mean ± SD of at least three independent experiments. Statistical significance was analyzed using Student’s *t* test (***p < 0.001). (c) Caseinolytic activities of both WT and *ΔhtrA* strains by casein-zymography. The *Gp*HtrA^WT^ protein was shown as the control. (d) The adhesion of wild-type or E-Cad KO NPTr cells by the WT or *ΔhtrA* strains (2 h of incubation at MOI of 100). Data were shown as mean ± SD from three independent experiments. Statistical significance was determined by using the two-way ANOVA (Sidak’s multiple comparisons test, ***p < 0.001, NS, not significant). (e) Western blot analysis of the E-cadherin cleavage and HtrA secretion in NPTr cells infected for 24 h with the WT strain and the *ΔhtrA* mutant (MOI of 100). GAPDH in whole cell lysates was shown as loading control. (f) E-cadherin expression (red) in NPTr cells with or without WT or *ΔhtrA* strains infection for 24 h. Scale bar, 20 μm. (g) The transmigration of both wild-type and E-Cad KO NPTr cells by WT or *ΔhtrA* strains (5 h of incubation at MOI of 2). The transmigrated bacteria was harvested in the basal chamber of Transwell. Data were shown as mean ± SD of at least three independent experiments. Statistical significance was analysis by using the two-way ANOVA (Sidak’s multiple comparisons test, ***p < 0.001, NS, not significant).

### *HtrA contributed to bacterial transmigration across porcine respiratory epithelial barrier* in vivo

**Figure 6.**
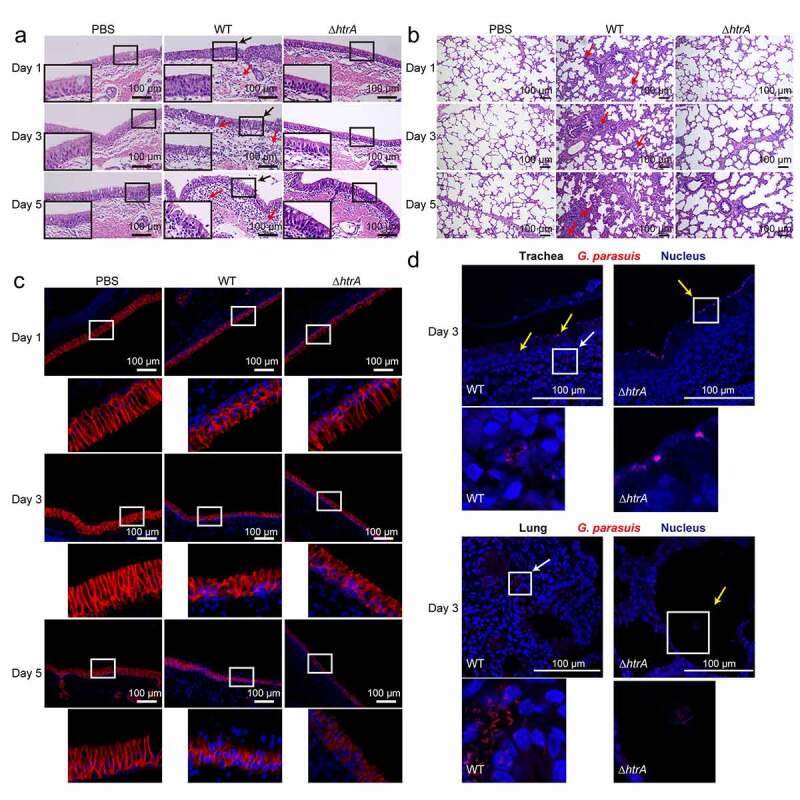
HtrA contributed to bacterial transmigration of the respiratory epithelial barrier

By comparing WT and the *ΔhtrA* strains, we next evaluated their transmigration and dissemination *in vivo* by intranasal challenge of experimental piglets. Bacterial loads in the nasal cavities, trachea, lungs, blood and joints at 1, 3, and 5 days post-infection (dpi) were determined. As shown in Table 2, the loads of the *ΔhtrA* strain was obviously lower in trachea and lungs, and undetectable in the joints and blood, compared with the WT strain. Noticeably at day 5, the WT strain could be detected in the blood and joint, suggesting the effective dissemination of the WT strain *in vivo* after intranasal inoculation (Table 2). Besides, the HE staining was performed to observed the tissue histopathology of trachea and lungs upon the infection. An apparent decrease of the cilia and detachment of the tracheal epithelium were observed, and a large number of inflammatory cells infiltrated and accumulated in the tracheal lamina propria as well as lung interstitium in piglets challenged with WT strain, while the trachea and lung challenged by *ΔhtrA* strain basically showed no such abnormalities (Figure 6(a,b)). Immunofluorescence staining also supported the significant disruption of E-cadherin in tracheal epithelial cells, as well as the epithelial detachment at day 5, in piglets challenged with WT, but not the *ΔhtrA* strain (Figure 6(c)). And the bacterial staining suggested that very few *ΔhtrA* bacteria were only found on the tracheal cilia or the alveoli of challenged piglets, and were absent in the tracheal lamina propria or the lung interstitium, however in contrast, the WT strain were much more detected in the respiratory epithelium, lamina propria or the lung interstitium (Figure 6(d)). The immunohistochemical analysis of the challenged trachea and lungs also confirmed this significantly different location of WT strain and the *ΔhtrA* mutant (Figure S6). These results conveyed that the HtrA protein in porcine respiratory bacterial pathogens largely facilitated bacterial penetration of the respiratory epithelial barrier and contributed to their proliferation and dissemination *in vivo*.

**Table 2.** Bacterial recovery from piglets infected with WT and *ΔhtrA* strains

Strain	Number of animals	Time of necropsy	Pulmonary infection			Systemic infection		
			Nasal cavities ^a^	Tracheal	Lung		Blood	Joint
WT	1	1 dpi	3	3	3		0	0
	1	3 dpi	3	3	3		0	0
	2	5 dpi	3/3	3/3	3/3		1/1	1/1
*ΔhtrA*	1	1 dpi	3	1	1		0	0
	1	3 dpi	3	2	2		0	0
	2	5 dpi	3/3	2/2	2/2		0/0	0/0

^a^Bacterial score of each animal in different tissue samples.

The trachea (a) and lung (b) sections from piglets receiving PBS, WT strain and *ΔhtrA* mutant challenge at day 1, 3, and 5. Red arrows indicated the infiltration of neutrophils and mononuclear cells in trachea lamina propria (a) or alveolar septum (b). The black arrows indicated the tracheal apical surface. The lower black panels were the magnified vision of the upper ones (a). Scale bar, 100 μm. (c) The tracheal sections showing the E-cadherin (red) disruption in piglets with the challenge of PBS, WT strain, and *ΔhtrA* mutant at day 1, 3, and 5. The lower images in each subpanel were the magnified vision in the white box of the upper ones. Scale bar, 100 μm. (d) The tracheal and lung sections showing the translocation of *G. parasuis* WT strain and the *ΔhtrA* mutant (red) at day 3 post infection. The panels below were the magnified vision of the boxed areas. White arrows indicated the transmigrated WT strain in the lamina propria and alveolar septum, and yellow arrows showed the challenged bacteria on the epithelial cells or in the alveolar space. Scale bar, 100 μm.

## Discussion

Mucosal defense is the critical mechanism in preventing respiratory bacterial pathogens colonization and exogenous antigens penetration of the epithelial barrier [47,48]. The coordinated activities of the respiratory mucosa involve the airway surface lining fluid which contains host defense peptides and mucus for the entrapment of inhaled particulates, cell-to-cell junctions between epithelial cells for restricting harmful substance transportion, and innate immune receptors and signaling pathways for the activation and recruitment of immune cells [10]. Airway epithelial cells, usually the first to encounter the invading microorganisms or inhaled substances, are the most abundant cell type in respiratory tract and are therefore important in the regulation of host defense [49]. Porcine respiratory bacterial pathogens, such as *G. parasuis, A. pleuropneumoniae, P. multocida*, and *B. bronchiseptica*, are the commensal or opportunistic pathogens in pig upper respiratory tract which are usually responsible for porcine respiratory disease complex. In swine, *B. bronchiseptica* and some toxin-producing *P. multocida* usually colonize respiratory mucosal surfaces, cause a chronic local infections called atrophic rhinitis [50]. However, under stress conditions, some of these pathogens like *G. parasuis* and *A. pleuropneumoniae* are able to break the airway epithelium and spread to multiple tissues, and cause severe systemic infections [7].

In the current work, we demonstrated that procine respiratory bacterial pathogens infection disrupted intercellular junctions and increased the permeability of monolayer epithelial cells for their transmigration. The adherence junction proteins, such as E-cadherin, are recognized the key components that maintain the epithelial barrier functions, and are also frequently impaired by the invading bacteria via the paracellular route [12,51]. Consequently, E-cadherin ectodomain shedding is frequently associated with the pathogenesis of acute lung injury [52]. A previous study has shown that porcine respiratory bacterial pathogen *G. parasuis* infection initiated the canonical Wnt/β-catenin signaling pathway leading to a disruption of E-cadherin [23], however, the specific mechanism and the significance of *G. parasuis* disrupting E-cadherin in airway epithelial cells were not very clear. Via CRISPR/Cas9 editing technology, the E-cadherin knockout cells were obtained and E-cadherin was confirmed to play important roles in maintaining airway epithelial barrier function and prevented the paracellular transmigration of respiratory bacteria. This suggested that E-cadherin was essential in preventing bacterial transmigration as well as systemic invasion by porcine bacterial pathogens.

Notably, the secretion of bacterial proteases is an efficient way for pathogens to colonize and cause pathology [6]. In human respiratory pathogens (*Klebsiella pneumoniae* and *Streptococcus pneumoniae*), the serine protease HtrA acted as an important secreted virulence factor in the development of pneumonia [53,54]. Previous studies have shown that the protease HtrA/DegQ protein was found in *G. parasuis* and *A. pleuropneumoniae* outer membrane vesicles [55,56], and we here demonstrated that HtrA protein was identified as a secreted serine protease of *G. parasuis in vitro* that directly targeted E-cadherin, and the serine site in the active center was critical for this E-cadherin proteolytic activity. Recent research shown that the HtrA protein plays a cruial role in inducing host immune response during *C. jejuni* infection of gnotobiotic mice [57], and we here found that *ΔhtrA* challenged-piglets displayed less accumulation of inflammatory cells infiltrated in the the tracheal lamina propria or lung interstitium, as compared to the WT strain challenged-piglets (Figure 6(a,b)).

The amino-terminal processing of HtrA strongly affects its secretion as well as regulatory activity [58]. Here, the analysis of *G. parasuis* bacterial lysates via Western blot indicated that *Gp*HtrA protein appeared as double bands, and the recombinant HtrA^WT^ or DegQ^WT^ protein also exhibited this double bands characteristic, suggesting the possible auto-processing of this protein in these bacteria. The previous study indicated that the amino-terminal auto-processing of HtrA could be affected by its LA loops that contribute to the integrity of the HtrA oligomer and the stability of the monomer [59]. Consistent with this concept, the entire amino-terminal of HtrA was deleted (*Gp*HtrA^ΔN^) that resulting in the loss of proteolytic activity and the disability to reduce the epithelial cells resistance (Figure 4(f), S4C and S4D). However interestingly, we observed that the small band of HtrA was significantly decreased when the inactive mutant was generated by substituting serine to alanine (S219A). This indicated a potential mechanism explaining the association of the amino-terminal auto-processing in HtrA with its proteolytic activity, which should be given further consideration in our future studies.

Additionally, we noticed the presence of host protease HtrA1 as well as the ADAMs in NPTr cells. However, the porcine respiratory bacterial-induced E-cadherin ectodomain shedding was independent of the host proteases HtrA1 and ADAM10, demonstrated by the ADAM10 inhibitor and HtrA1 siRNA approaches. These data further supported the involvement of bacterial determinants in mediating E-cadherin cleavage. By using HtrA-deleted *G. parasuis*, we moreover found HtrA-mediated E-cadherin cleavage in host cells largely contributed to bacterial paracellular transmigration. And this bacterial transmigration differences between HtrA-deleted and WT strains disappeared in the E-Cad KO NPTr cells *in vitro*. Importantly, deletion of *htrA* gene did not impair the growth of *G. parasuis* at 37°C, but significantly attenuated the adherence of *ΔhtrA* strain to both wild-type and E-Cad KO NPTr cells. This imply that the role of HtrA in mediating bacterial adherence had no relationship to E-cadherin. Anyway, it will be interesting to study whether there are other bacterial proteases that involving the cleavage of E-cadherin and how they act to help bacteria overcome the epithelial barrier in the respiratory tract. The solution of these problems will help to better understand the molecular mechanisms of porcine respiratory pathogens breaking through the airway epithelial barrier to cause disease.

## Supplementary Material

Supplemental Material

## Data Availability

Data sharing is not applicable to this article as no new data were created or analyzed in this study.
