# Supplementary material for: Cleavage of E-cadherin by porcine respiratory bacterial pathogens facilitates airway epithelial barrier disruption and bacterial paracellular transmigration
Source: Virulence. 2021 Sep 5;12(1):2296–313. doi: 10.1080/21505594.2021.1966996 (PMC8425755; doi:10.1080/21505594.2021.1966996)
Supplement: Supplemental Material [file KVIR_A_1966996_SM6607.zip › suppl/Supplementary Tables.docx]

**Supplementary Tables**

**Table S1 Bacterial strains and plasmids used in this study.**

| Strains and plasmids | Characteristics | Source |
| --- | --- | --- |
| BL21 (DE3) | *F^-^ ompT hsdS_B_(r_B_^-^ m_B_^-^) gal dcm* | Takara |
| DH5α | *sup*E44 *Δlac*U169 (φ80 *lac*Z*ΔM1*) *hsd*R17 *end*A1 *gyr*A96 thi-1 *rel*A1 | Takara |
| *G. parasuis* clinical isolate CF7066 | Serotype 5 | This study |
| *A. pleuropneumoniae* reference strain S4074 | Serotype 1 | This study |
| *P. multocida* clinical isolate HB03 | Serotype A | This study |
| *B. bronchiseptica* clinical isolate Bb1701079 | Unknown | This study |
| *G. parasuis* CF7066*ΔhtrA* | Serotype 5 | This study |
| pK18mobsacB | Suicide and narrow-broad-host vector, Kan resistance |  |
| pK18mobsacB-*htrA*-UKD | A 1929 bp overlap fragment containing Kan resistance, the upstream and downstream of the *htrA* gene in pK18mobsacB | This study |
| pET28a | *E. coli* expression vector, Kan resistance | Novagen |
| pET28a-HtrA^WT^/DegQ^WT^ | pET28a containing wild-type HtrA/DegQ | This study |
| pET28a-HtrA^SA^/DegQ^SA^ | pET28a containing inactive mutant HtrA/DegQ by exchanging Serine to Alanine | This study |
| pET28a-HtrA^ΔN^ | pET28a containing N-terminal deletion of wild-type HtrA | This study |
| p3×FLAG-CMV-14 | Mammalian cell expression vector, Amp resistance | Sigma-Aldrich |
| p3×FLAG-CMV-14-NTF | A 1995 bp extracellular domain of porcine E-cadherin in p3×FLAG-CMV-14 | This study |

**Table S2 Primers related to deletion *htrA* gene in this study.**

| Primers | Primer sequences (5′ to 3′) ^a^ |
| --- | --- |
| *htrA*-upstream-F/R | CCGGAATTCACCGCTTGTTAATGCTGGGTCAGTTCAAA |
|  | **TTTTTATCTTGTGCAATG**AAAGTTTTGCGATACTATCATTT |
| *htrA*-downstream-F/R | **AATCAGAATTGGTTAATT**GTGATTATCCTTACTAAATTTATGAT |
|  | CGCGGATCCACAAGCGGTCCAAGGATAGGACCAAAGAT |
| *Kan*-F/R | CATTGCACAAGATAAAAATA |
|  | CAATTAACCAATTCTGATTA |
| Internal-*htrA*-F/R | GGTTCTCCTCGTCAGTTT |
|  | TTGAGTTCACCGCCTTTA |

^a^ restriction recognition sites are underlined, overlapped sequences are in bold.

**Table S3 Proteins in negatively stained bands of *G. parasuis* and identified by mass spectrometry.**

| Protein accession number | Protein Description | L-1 ^a^ | M-2 | S-3 |
| --- | --- | --- | --- | --- |
| A0A076V055 | Elongation factor Tu | 28.61 | 14.18 | 74.18 |
| U4RQS6 | Trigger factor | 1.39 |  | 69.61 |
| B8F6T9 | Trigger factor | 1.39 |  | 69.37 |
| D9IQX9 | Outer membrane protein P2 | 61.60 | 58.29 | 53.31 |
| U4RMT8 | 3-oxoacyl-(Acyl carrier protein) synthase I | 1.73 |  | 61.48 |
| A0A143CF36 | Cysteine desulfurase IscS | 3.45 | 2.22 | 58.37 |
| D3JWF0 | Outer membrane protein P2 | 58.13 | 47.93 | 42.98 |
| A0A5C1NYC3 | DNA-directed RNA polymerase subunit beta | 56.69 | 7.22 | 2.59 |
| A0A017QL78 | Transketolase | 17.66 | 54.04 | 10.03 |
| A0A5C1NUY5 | Chaperone protein DnaK | 1.11 | 53.55 | 7.27 |
| A0A5C1NZH7 | Acetate kinase |  | 3.26 | 52.13 |
| A0A5C1P0S9 | ABC transporter substrate-binding protein | 1.69 | 3.39 | 51.98 |
| U4RQ02 | DNA-directed RNA polymerase subunit beta | 51.12 | 8.49 | 1.86 |
| A0A5C1NXI6 | 30S ribosomal protein S1 | 9.01 | 45.05 | 5.95 |
| P80369 | Outer membrane protein P2 (Fragment) | 47.62 | 47.62 | 47.62 |
| U4S9Y0 | Phosphoglycerate kinase |  |  | 46.55 |
| U4SPY9 | ABC transporter substrate-binding protein |  |  | 46.14 |
| A0A084EH38 | Fumarate reductase flavoprotein subunit | 1.85 | 45.45 | 6.90 |
| B8F8E8 | Protein HflK |  |  | 45.30 |
| A0A084EV83 | 50S ribosomal protein L2 | 20.88 | 33.70 | 19.41 |
| B7SY85 | OmpA | 16.89 | 19.89 | 41.42 |
| A0A143CGF2 | 50S ribosomal protein L21 | 43.69 | 17.48 | 27.18 |
| A0A145QRZ4 | Transketolase | 12.13 | 43.56 | 6.14 |
| U4SJK6 | Acetate kinase |  |  | 43.36 |
| A0A5C1NWS6 | Serine hydroxymethyltransferase |  | 1.90 | 42.62 |
| A0A084EZL0 | Queuine tRNA-ribosyltransferase |  |  | 42.60 |
| A0A084ECS1 | ATP-dependent Clp protease ATP-binding subunit ClpX | 2.16 |  | 42.55 |
| A0A384XSK5 | Chromosome partition protein MukB | 42.20 |  |  |
| A0A068FL62 | Outer membrane protein P5 (Fragment) | 17.77 | 16.33 | 38.97 |
| A0A084EEZ9 | Transcription termination factor Rho |  |  | 41.19 |
| B8F774 | ATP synthase subunit beta |  |  | 40.26 |
| A0A084F0W6 | Biotin carboxylase |  |  | 40.04 |
| U4RMD0 | Phosphoglycerate kinase |  |  | 39.64 |
| A0A145QQM6 | Deoxyguanosinetriphosphate triphosphohydrolase-like protein |  |  | 39.09 |
| A0A1T0ACH2 | NAD(P) transhydrogenase subunit alpha |  |  | 39.02 |
| A0A5C1NY50 | Phosphoglucosamine mutase |  |  | 38.96 |
| A0A5C1P1E8 | UDP-N-acetylglucosamine 1-carboxyvinyltransferase |  |  | 38.92 |
| A0A017QIW6 | Bifunctional DNA-binding transcriptional repressor/ NMN adenylyltransferase | 2.36 |  | 38.77 |
| B8F492 | Polyribonucleotide nucleotidyltransferase | 5.36 | 37.80 | 3.10 |
| B8F7C2 | Proline-tRNA ligase |  | 38.53 |  |
| B8F6X4 | tRNA uridine 5-carboxymethylaminomethyl modification enzyme MnmG |  | 38.31 |  |
| A0A145QU86 | Spermidine/putrescine import ATP-binding protein PotA |  | 2.43 | 38.11 |
| B8F6L7 | Aminopeptidase B |  |  | 37.96 |
| U4RRZ3 | 30S ribosomal protein S3 | 37.61 | 16.67 | 9.83 |
| B8F7Z4 | Elongation factor G | 8.00 | 36.71 | 3.00 |
| B8F8Q3 | Isocitrate dehydrogenase [NADP] |  |  | 36.63 |
| B8F7A7 | NADH dehydrogenase |  |  | 35.60 |
| A0A084EH10 | Isocitrate dehydrogenase [NADP] |  |  | 34.94 |
| A0A143CFH7 | Adenylosuccinate synthetase |  |  | 34.72 |
| B8F7B9 | Pyridoxal kinase |  | 3.64 | 34.55 |
| A0A5C1P165 | Fatty-acid oxidation protein subunit alpha | 21.25 | 29.04 | 1.42 |
| A0A5C1NXG8 | Coproporphyrinogen-III oxidase |  |  | 34.07 |
| U4SCQ5 | 50S ribosomal protein L19 | 20.69 |  | 12.93 |
| B8F3Y8 | Phosphoenolpyruvate carboxylase | 33.33 | 2.96 |  |
| A0A5C1P0J9 | Phosphoenolpyruvate carboxylase | 33.22 | 2.96 |  |
| A0A1S9ZYC5 | Glutamate-tRNA ligase |  |  | 32.99 |
| A0A084ELK7 | GTP pyrophosphokinase | 11.56 | 29.66 |  |
| A0A084EW64 | GTP-binding protein TypA | 2.93 | 32.20 |  |
| A0A084EV29 | Uridine phosphorylase |  | 32.16 | 4.31 |
| B8F502 | Ribosome-binding ATPase YchF |  |  | 31.68 |
| U4RXC7 | Oligopeptide ABC transporter periplasmic binding protein | 9.30 | 13.09 | 28.65 |
| B8F5M0 | Porphoryin biosynthesis protein HemY |  |  | 31.49 |
| A0A5C1NXS7 | DUF945 domain-containing protein |  |  | 31.21 |
| B8F639 | Protein RecA |  |  | 31.18 |
| A0A084EAM4 | Fumarate hydratase class II |  |  | 31.03 |
| A0A5C1P284 | Adenylosuccinate lyase |  |  | 30.77 |
| A0A084F0S4 | Ketol-acid reductoisomerase (NADP(+)) |  |  | 30.22 |
| A0A143CHF4 | Ketol-acid reductoisomerase (NADP(+)) |  |  | 30.02 |
| U4RU42 | Bifunctional prephenate dehydratase/chorismate mutase |  |  | 29.64 |
| U4S2L6 | Transcription termination/antitermination protein NusA |  | 29.61 |  |
| A0A5C1NYA9 | Carboxy terminal-processing peptidase | 19.91 | 25.75 | 4.34 |
| B8F3K4 | Bifunctional protein GlmU |  |  | 29.36 |
| A0A017QKG0 | Aminopeptidase B |  |  | 29.14 |
| U4RWX9 | Dihydrolipoyllysine-residue succinyltransferase component of 2-oxoglutarate dehydrogenase complex |  |  | 29.14 |
| A0A084EWC9 | 50S ribosomal protein L20 | 29.06 |  | 7.69 |
| U4S0B4 | ATP-dependent RNA helicase RhlB | 2.71 |  | 29.06 |
| U4RLM7 | Enolase |  |  | 28.90 |
| B8F3T2 | GMP synthase (glutamine-hydrolyzing) | 2.68 | 15.11 | 24.67 |
| A0A5C1NVS5 | Polysaccharide biosynthesis tyrosine autokinase |  | 28.63 |  |
| U4RLJ6 | Outer membrane protein | 7.36 | 2.16 | 27.06 |
| A0A143CER6 | Exopolyphosphatase |  |  | 28.26 |
| A0A143CFV2 | 30S ribosomal protein S9 | 10.69 | 28.24 | 28.24 |
| A0A5C1P1C7 | Threonine-tRNA ligase | 12.75 | 23.48 | 0.93 |
| A0A084EEW8 | 60 kDa chaperonin | 6.58 | 14.44 | 20.11 |
| A0A5C1P0F5 | Alanine-tRNA ligase | 27.43 | 3.20 |  |
| A0A143CFG4 | Catalase | 23.03 | 12.86 | 12.03 |
| A0A084EYP9 | Alanine-tRNA ligase | 27.35 | 3.20 |  |
| A0A5C1P1D4 | Acetyltransferase component of pyruvate dehydrogenase complex | 22.41 | 23.89 | 6.85 |
| A0A017QL93 | S-adenosylmethionine synthase |  |  | 26.96 |
| A0A143CES6 | Methionine-tRNA ligase | 16.52 | 24.85 |  |
| U4S2Z9 | Uncharacterized protein | 26.39 |  |  |
| B8F496 | LPS-assembly protein LptD | 13.87 | 20.67 | 4.80 |
| A0A5C1NXG9 | Glutathione-disulfide reductase |  |  | 26.10 |
| A0A145QTK4 | 6-phosphogluconate dehydrogenase, decarboxylating | 8.68 | 4.96 | 23.97 |
| B8F4A3 | Dihydrolipoyl dehydrogenase | 10.13 | 11.18 | 20.04 |
| B8F6L1 | Glutathione reductase |  |  | 25.88 |
| U4RVU0 | RNA polymerase sigma factor RpoD | 18.52 | 22.06 |  |
| C9WEY6 | Outer membrane protein P5 (Fragment) | 9.69 | 9.69 | 25.51 |
| B8F3B2 | UDP-N-acetylmuramoyl-tripeptide--D-alanyl-D-alanine ligase |  |  | 25.43 |
| A0A084EWR4 | D-ribose-binding periplasmic protein |  |  | 25.14 |
| B8F8M2 | 3-hydroxydecanoyl-(acyl-carrier-protein) dehydratase |  | 19.89 | 25.00 |
| A0A084EWU3 | Glucose-1-phosphate adenylyltransferase |  |  | 24.71 |
| A0A145QXE7 | Serine-tRNA ligase |  | 1.84 | 24.65 |
| U4RWI3 | Outer membrane protein assembly factor BamA | 1.24 | 23.60 | 8.70 |
| U4RXK3 | ABC transporter ATP-binding protein | 19.94 | 8.48 | 1.73 |
| A0A143CEL4 | Chromosome partition protein MukF |  |  | 24.15 |
| A0A5C1P211 | Glutamate-1-semialdehyde 2,1-aminomutase |  |  | 24.12 |
| U4RTM9 | Ribonucleotide-diphosphate reductase subunit alpha | 3.51 | 5.45 | 24.08 |
| A0A084EX70 | Putative ABC transporter ATP-binding protein | 21.35 | 6.59 |  |
| A0A017QHK2 | Putative acridine efflux pump AcrA, RND efflux membrane-fusion protein |  |  | 23.68 |
| A0A1S9ZZI4 | Outer membrane protein assembly factor BamA | 1.24 | 21.99 | 7.95 |
| A0A1T0AAR0 | Chaperone protein HtpG | 20.38 | 18.95 | 1.59 |
| A0A084EY70 | Aminopeptidase N | 23.36 | 9.78 |  |
| A0A084EWJ4 | Na(+)-translocating NADH-quinone reductase subunit A |  |  | 23.21 |
| B8F5X1 | Aspartate ammonia-lyase |  |  | 23.16 |
| A0A084ELJ7 | Outer membrane lipid asymmetry maintenance protein MlaD |  | 23.08 |  |
| A0A143CHL3 | 30S ribosomal protein S13 | 22.03 | 7.63 | 15.25 |
| A0A1T0A1H9 | Threonine synthase |  |  | 22.88 |
| A0A143CGM2 | DNA polymerase III subunit alpha | 22.71 |  |  |
| A0A5C1P317 | Sialidase |  | 22.54 |  |
| A0A084EZH4 | 3-phosphoshikimate 1-carboxyvinyltransferase |  |  | 22.43 |
| A0A5C1NWU7 | 3-phosphoshikimate 1-carboxyvinyltransferase |  |  | 22.20 |
| A0A5C1NYT4 | Formate C-acetyltransferase | 9.82 | 20.28 | 2.20 |
| U4RS51 | tRNA modification GTPase MnmE |  |  | 21.90 |
| B8F6P7 | 30S ribosomal protein S4 | 14.08 | 14.56 | 21.84 |
| A0A5C1P2J6 | tRNA modification GTPase MnmE |  |  | 21.68 |
| A0A1T0A6W9 | Succinate-CoA ligase [ADP-forming] subunit beta |  |  | 21.65 |
| A0A5C1NY37 | 50S ribosomal protein L15 | 16.67 | 4.86 | 17.36 |
| C9WEZ0 | Outer membrane protein P5 (Fragment) |  | 6.30 | 21.26 |
| A0A5C1NWA4 | Glutamate synthase large subunit | 21.24 |  |  |
| A0A5C1NXN6 | Phosphate acetyltransferase | 16.32 | 18.42 | 6.05 |
| A0A084EY87 | NAD(P) transhydrogenase subunit beta |  |  | 21.23 |
| B8F4G5 | Uncharacterized protein |  |  | 20.89 |
| A0A5C1NYJ8 | Phosphoglycerate dehydrogenase |  |  | 20.73 |
| B8F6T8 | Putative rhodanese-related sulfurtransferase |  | 20.67 |  |
| A0A5C1P2E5 | PTS maltose transporter subunit IIBC |  | 1.90 | 20.51 |
| B8F6L2 | Uncharacterized protein |  |  | 20.47 |
| A0A384XDD0 | Transcriptional regulator |  |  | 20.47 |
| A0A1T0AFS7 | Elongation factor 4 | 4.36 | 20.44 |  |
| U4RRZ2 | Periplasmic serine endoprotease DegP-like |  |  | 20.22 |
| A0A084EAA3 | Uncharacterized protein |  |  | 20.22 |
| B8F3L6 | Protein translocase subunit SecA | 20.13 | 1.45 |  |
| A0A084EXW2 | Argininosuccinate synthase |  | 3.39 | 20.09 |
| A0A5C1P2L2 | Ribulose-phosphate 3-epimerase |  | 20.09 |  |
| A0A145QGU2 | DNA helicase |  | 20.08 |  |
| A0A084EH54 | Glycerol-3-phosphate acyltransferase | 18.87 | 7.03 | 2.22 |
| A0A017QGP9 | Exodeoxyribonuclease VII large subunit | 3.27 |  | 19.89 |
| B8F7N1 | Type III restriction-modification system EcoPI enzyme mod |  | 19.84 | 3.59 |
| A0A084F0F6 | Delta-aminolevulinic acid dehydratase | 7.65 |  | 19.71 |
| A0A084EW18 | tRNA-specific 2-thiouridylase MnmA |  |  | 19.58 |
| A0A1T0A1J9 | Glutamate synthase (Ferredoxin) subunit alpha | 19.49 |  |  |
| A0A143CFM8 | DNA-directed RNA polymerase subunit delta |  | 19.44 | 9.09 |
| A0A017QKG3 | Membrane protein insertase YidC |  | 17.95 | 1.47 |
| D9IQT1 | Outer membrane protein P1 |  | 5.86 | 19.37 |
| A0A5C1P322 | Alpha-1,4 glucan phosphorylase | 19.29 |  |  |
| B8F8H3 | Xaa-Pro dipeptidase |  |  | 19.09 |
| U4S2T0 | Cytochrome D ubiquinol oxidase subunit I |  |  | 18.99 |
| U4S208 | Inosine-5'-monophosphate dehydrogenase (Fragment) | 8.93 | 3.57 | 12.50 |
| A0A084EV11 | Mannose-6-phosphate isomerase |  |  | 18.86 |
| A0A5C1P0V1 | Single-stranded DNA-binding protein | 18.79 | 6.06 |  |
| A0A143CGA4 | Glutamate synthase large subunit | 18.68 |  |  |
| A0A5C1NWV1 | Glycine-tRNA ligase beta subunit | 9.08 | 15.74 |  |
| A0A143CE51 | Ornithine carbamoyltransferase |  |  | 18.15 |
| A0A5C1P2J3 | ABC transporter substrate-binding protein |  |  | 18.08 |
| A0A5C1NYY4 | DNA topoisomerase 1 | 16.78 | 4.25 |  |
| A0A5C1NZE6 | M13 family metallopeptidase | 17.19 |  |  |
| A0A5C1P3C4 | Ribonuclease E | 16.36 | 6.63 | 0.62 |
| A0A377INC2 | ABC transporter ATP-binding protein |  | 15.22 | 9.06 |
| Q0QE60 | RpoA (Fragment) | 7.28 |  | 12.58 |
| A0A5C1P0A9 | ABC transporter ATP-binding protein | 2.70 | 16.67 |  |
| A0A084EPW4 | Chromosomal replication initiator protein DnaA |  |  | 16.67 |
| A0A1S9ZYA7 | Beta sliding clamp |  | 2.45 | 16.62 |
| B8F535 | Histidine--tRNA ligase |  |  | 16.23 |
| U4RNH4 | Triosephosphate isomerase (Fragment) |  |  | 16.00 |
| A0A084EZX1 | Signal recognition particle protein |  | 1.52 | 14.35 |
| A0A1T0A1K9 | Riboflavin biosynthesis protein RibBA |  |  | 15.79 |
| U4S9V2 | Citrate synthase |  |  | 15.73 |
| A0A5C1P1J7 | Pitrilysin | 4.18 | 15.00 | 6.43 |
| B8F6P8 | 30S ribosomal protein S11 | 9.30 |  | 15.50 |
| A0A143CGE0 | Bifunctional aspartokinase/homoserine dehydrogenase | 15.48 | 3.56 | 2.21 |
| A0A5C1P3A1 | Peptidylprolyl isomerase |  | 15.38 |  |
| A0A5C1NZ91 | Multifunctional fusion protein |  |  | 15.13 |
| A0A145QPM3 | DNA repair protein RadA |  |  | 15.07 |
| A0A5C1NYN2 | Ribosomal RNA large subunit methyltransferase K/L |  | 15.03 |  |
| A0A5C1NYC2 | Phosphoribosylformylglycinamidine synthase | 15.02 |  |  |
| A0A084ELI9 | DNA starvation/stationary phase protection protein | 14.91 | 10.56 | 10.56 |
| B8F5Z2 | 30S ribosomal protein S15 | 14.61 |  |  |
| B8F8E4 | Type III restriction-modification system methyltransferase (Adenine-specific)/adenine specific DNA methylase Mod | 3.15 | 12.60 |  |
| U4RRR6 | Gamma-glutamyl phosphate reductase |  |  | 14.42 |
| A0A5C1P0J0 | Pyruvate dehydrogenase E1 component | 10.62 | 11.19 | 1.13 |
| A0A143CHB6 | 50S ribosomal protein L6 | 6.21 | 6.21 | 7.91 |
| A0A145QY65 | Exoribonuclease 2 |  | 13.81 |  |
| A0A1T0A6Q6 | 2-oxoglutarate dehydrogenase E1 component | 13.80 | 0.96 |  |
| B8F4W7 | Type I restriction enzyme specificity protein HsdS |  |  | 13.73 |
| B8F772 | ATP synthase subunit alpha | 3.90 | 9.75 | 9.16 |
| U4RZI6 | Pyruvate dehydrogenase E1 component | 9.72 | 10.28 | 1.13 |
| A0A5C1P1W2 | Na(+)-translocating NADH-quinone reductase subunit F |  |  | 13.17 |
| A0A084EX13 | Magnesium transport protein CorA |  |  | 13.08 |
| A0A084EJL3 | Isoleucine--tRNA ligase | 10.77 | 4.37 | 0.64 |
| B8F858 | 50S ribosomal protein L28 | 12.82 |  |  |
| A0A5C1NZD1 | DNA polymerase III subunit gamma/tau |  | 12.80 | 1.34 |
| U4RYE2 | Pyruvate kinase domain protein | 12.64 |  |  |
| A0A084ETK4 | Protease 3 | 3.67 | 11.84 | 5.31 |
| A0A143CHF2 | Mechanosensitive ion channel protein MscS | 12.54 |  |  |
| A0A084F0Y1 | Bifunctional aspartokinase/homoserine dehydrogenase | 12.53 | 4.91 | 3.56 |
| B8F3B4 | UDP-N-acetylmuramoylalanine--D-glutamate ligase |  |  | 12.50 |
| A0A145QDI9 | Ion-translocating oxidoreductase complex subunit C |  | 12.46 |  |
| B8F6G8 | Bifunctional protein PutA | 11.89 | 2.33 |  |
| A0A084ELH2 | Gamma-glutamylputrescine oxidoreductase |  |  | 11.81 |
| A0A5C1P263 | Fe(3+) ABC transporter substrate-binding protein | 5.28 | 4.40 | 6.74 |
| A0A017QJH6 | 50S ribosomal protein L1 | 11.35 | 5.24 | 5.24 |
| A0A084F008 | CoA-acylating methylmalonate-semialdehyde dehydrogenase |  |  | 11.33 |
| B8F7D2 | 50S ribosomal protein L1 | 4.93 |  | 6.34 |
| A0A143CFV5 | DNA topoisomerase 4 subunit B | 8.85 | 8.53 |  |
| A0A143CFF0 | 6-phosphofructokinase |  |  | 11.18 |
| A0A084EW05 | UDP-N-acetylmuramate--L-alanine ligase |  |  | 11.16 |
| A0A084EZT1 | N-acetyl-anhydromuranmyl-L-alanine amidase |  |  | 11.11 |
| A0A084EF23 | Peptide chain release factor 1 |  |  | 11.11 |
| A0A084EAB7 | Lactate utilization protein B |  |  | 10.90 |
| A0A084EY60 | ATP-dependent 6-phosphofructokinase |  |  | 10.90 |
| A0A084EZ41 | Tyrosine-tRNA ligase |  |  | 10.86 |
| A0A5C1NZB8 | Protease 4 | 10.79 |  |  |
| B8F663 | RNA-binding protein Hfq | 10.75 |  |  |
| B8F5T5 | 30S ribosomal protein S18 | 10.67 |  | 10.67 |
| A0A5C1P119 | Peptidyl-prolyl cis-trans isomerase | 10.64 | 4.79 | 10.64 |
| A0A5C1P0A6 | ATP-dependent RNA helicase DeaD |  | 10.45 |  |
| A0A084EFR0 | Periplasmic nitrate reductase | 4.59 | 5.56 | 1.33 |
| A0A084F120 | Putative uroporphyrinogen-III C-methyltransferase |  | 5.30 | 7.51 |
| A0A084EX68 | Molybdopterin molybdenumtransferase |  |  | 10.24 |
| A0A084EVU2 | Chaperone protein ClpB |  | 10.15 |  |
| A0A084EV17 | Protein-export protein SecB | 5.36 |  | 10.12 |
| A0A145QQ20 | ABC transporter substrate-binding protein |  |  | 10.06 |
| A0A5C1P0C5 | Formate dehydrogenase-N subunit alpha | 7.93 | 6.46 |  |
| A0A084EV75 | Metalloprotease TldD |  |  | 9.77 |
| A0A084EY07 | Cytochrome c551 peroxidase |  |  | 9.68 |
| A0A145QE51 | ATP-dependent RNA helicase HrpA | 9.50 |  |  |
| A0A017QHG1 | ATP-dependent RNA helicase HrpA | 9.39 |  |  |
| A0A145QVI1 | Glutamyl-tRNA reductase |  |  | 9.20 |
| A0A5C1NXJ6 | Peptide ABC transporter substrate-binding protein |  |  | 9.16 |
| A0A017QJX0 | Lysozyme (Fragment) |  |  | 8.97 |
| B8F6Q9 | 50S ribosomal protein L5 | 8.94 | 4.47 | 4.47 |
| A0A017QLR8 | 4-hydroxy-3-methylbut-2-en-1-yl diphosphate synthase (flavodoxin) |  |  | 8.94 |
| U4S3C4 | Nitrate/sulfonate/bicarbonate ABC transporter periplasmic protein | 1.55 | 1.55 | 8.76 |
| B8F3Q1 | Multifunctional CCA protein |  |  | 8.75 |
| B8F891 | Cell division protein FtsP |  |  | 8.74 |
| U4S0U5 | Cell division protein FtsA |  |  | 8.71 |
| U4RV16 | 3-oxoacyl-[acyl-carrier-protein] reductase |  |  | 8.71 |
| A0A084EW74 | Iron-sulfur cluster assembly scaffold protein IscU | 8.66 |  |  |
| B8F6R1 | 50S ribosomal protein L14 | 8.65 | 8.65 |  |
| A0A145R0T4 | Aerobic respiration control sensor protein |  | 8.60 |  |
| A0A145QI54 | Alpha-D-phosphohexomutase |  | 2.72 | 8.33 |
| U4RPN0 | Pseudouridine synthase |  |  | 8.31 |
| A0A1T0A833 | Argininosuccinate lyase |  |  | 8.30 |
| A0A084ELF8 | Protease 4 | 8.21 |  |  |
| B8F690 | ATP-dependent protease ATPase subunit HslU |  |  | 8.18 |
| U4S263 | Putative tubulin binding protein | 8.15 |  |  |
| B8F7Y2 | Toll-interleukin receptor | 8.10 |  |  |
| Q2Y2P5 | Superoxide dismutase (Fragment) |  |  | 8.05 |
| A0A143CIQ8 | Glutamine--fructose-6-phosphate aminotransferase | 4.75 | 1.15 | 4.59 |
| A0A5C1P0G5 | Dihydroxy-acid dehydratase | 1.64 |  | 8.02 |
| A0A017QIY1 | Penicillin-binding protein 1A | 6.70 |  | 1.29 |
| A0A017QI94 | Quinone-dependent D-lactate dehydrogenase | 1.95 | 3.91 | 6.04 |
| A0A5C1NXT1 | Pseudouridine synthase |  | 7.96 |  |
| A0A5C1NX85 | Glutamine-tRNA ligase | 1.61 | 7.89 |  |
| A0A145QSD5 | Oligopeptide ABC transporter substrate-binding protein OppA |  | 4.59 | 3.30 |
| A0A5C1NXA6 | Aminoacyl-histidine dipeptidase |  |  | 7.85 |
| A0A084ELG8 | Fructose-bisphosphate aldolase | 4.74 |  | 3.06 |
| B8F8D8 | Uncharacterized protein | 7.69 |  |  |
| A0A143CF96 | Uncharacterized protein |  | 7.69 |  |
| A0A084EZ21 | 30S ribosomal protein S7 |  |  | 7.69 |
| B8F376 | Tol-Pal system protein TolB |  |  | 7.67 |
| A0A5C1NY32 | Acetyl-CoA C-acyltransferase |  |  | 7.60 |
| A0A084EPV5 | ATP-dependent DNA helicase RecG |  | 7.50 |  |
| A0A084EF43 | MPN domain-containing protein | 7.45 |  |  |
| A0A084EXB5 | Bifunctional protein folC |  |  | 7.41 |
| A0A5C1P0U1 | Cell division protein FtsZ |  |  | 7.35 |
| B8F3R5 | Lysine--tRNA ligase |  | 1.86 | 7.25 |
| A0A143CEC6 | DNA gyrase subunit B | 3.70 | 4.69 |  |
| U4RY29 | Uncharacterized protein | 7.14 |  |  |
| A0A5C1P2A0 | UDP-N-acetylmuramate--L-alanyl-gamma-D-glutamyl-meso-2,6-diaminoheptandioate ligase |  |  | 7.14 |
| A0A084EAC1 | Glutamate 5-kinase |  |  | 7.10 |
| A0A5C1NZR1 | Translation initiation factor IF-2 | 4.73 | 3.78 | 2.36 |
| A0A1S9ZXN1 | Baseplate J protein |  | 7.08 |  |
| Q3YJ39 | Aconitase putative (Fragment) | 3.39 | 4.65 |  |
| A0A143CGS8 | Ribosomal protein S12 methylthiotransferase RimO |  |  | 7.00 |
| B8F5M8 | Lon protease | 6.88 |  |  |
| A0A084EF33 | Uncharacterized protein |  |  | 6.86 |
| A0A143CFT1 | Metalloprotease |  | 6.85 |  |
| A0A145QRG8 | Phosphoenolpyruvate-protein phosphotransferase | 4.89 | 5.41 | 1.40 |
| U4RRZ8 | Leucine-tRNA ligase | 6.74 |  |  |
| A0A5C1NXS6 | 16S rRNA (Cytosine(967)-C(5))-methyltransferase RsmB |  |  | 6.71 |
| B8F7G6 | Outer membrane antigenic lipoprotein B |  |  | 6.65 |
| B8F362 | Chaperone protein HscA homolog |  |  | 6.63 |
| A0A143CIA8 | 50S ribosomal protein L16 | 6.62 | 6.62 |  |
| A0A084F0T3 | Pseudouridine synthase |  | 6.58 |  |
| A0A084EVX0 | Pyruvate formate-lyase-activating enzyme | 6.50 |  |  |
| B8F5Q0 | Guanosine polyphosphate pyrophosphohydrolase/synthetase | 6.44 |  |  |
| A0A084EY21 | CDP-diacylglycerol-serine O-phosphatidyltransferase |  |  | 6.37 |
| A0A5C1P301 | 50S ribosomal protein L22 |  |  | 6.36 |
| A0A145R1Y1 | Lipoprotein |  |  | 6.09 |
| A0A084ELH6 | Dual-specificity RNA methyltransferase RlmN |  |  | 5.96 |
| A0A084EY20 | High-affinity zinc uptake system protein znuA | 5.77 | 2.88 |  |
| A0A084F0R5 | Signal recognition particle receptor FtsY |  | 5.71 |  |
| A0A084EW11 | UDP-N-acetylmuramoyl-L-alanyl-D-glutamate--2,6-diaminopimelate ligase |  |  | 5.65 |
| A0A5C1P0V5 | Asparagine--tRNA ligase |  | 1.50 | 5.57 |
| A0A084EUY6 | Peptidase T |  |  | 5.53 |
| A0A084EVE2 | Peptidoglycan glycosyltransferase MrdB |  |  | 5.45 |
| B8F7L3 | DNA-binding transcriptional repressor FabR | 5.37 |  |  |
| A0A084F154 | Carbamoyl-phosphate synthase large chain | 5.35 |  |  |
| A0A5C1P1X6 | Penicillin-binding protein 1B | 5.29 |  |  |
| A0A145R1L0 | Lipopolysaccharide assembly protein B |  |  | 5.29 |
| A0A084EY18 | Membrane-bound lytic murein transglycosylase F |  |  | 5.29 |
| A0A084EW15 | Transcriptional regulator MraZ | 5.26 |  |  |
| B8F807 | Valine--tRNA ligase | 5.23 | 1.00 |  |
| A0A5C1NY80 | Class I adenylate cyclase | 3.16 | 4.37 |  |
| T1RQ56 | Aminodeoxychorismate lyase | 5.18 |  |  |
| B8F5G0 | 23S rRNA (uracil(1939)-C(5))-methyltransferase RlmD |  |  | 5.05 |
| A0A084EZJ5 | Sensor protein CpxA |  |  | 5.02 |
| A0A084EH34 | Glyceraldehyde-3-phosphate dehydrogenase |  | 2.36 | 5.01 |
| A0A143CFT4 | Autotransporter domain-containing protein | 4.92 |  |  |
| A0A084EWW2 | Single-stranded-DNA-specific exonuclease recJ | 4.86 |  | 1.56 |
| A0A143CH61 | 50S ribosomal protein L3 | 4.81 |  | 4.81 |
| A0A143CEA2 | UvrABC system protein A | 4.14 | 0.64 |  |
| U4RWB4 | Prophage CP4-57 integrase |  |  | 4.69 |
| A0A377IJI5 | Glycerate dehydrogenase | 4.62 | 4.62 | 4.62 |
| A0A084EVU4 | DNA polymerase I | 2.20 | 3.88 |  |
| U4S2C6 | Binding--dependent transport system inner membrane component family protein (Fragment) |  |  | 4.55 |
| A0A384XRN5 | Uncharacterized protein |  | 4.50 |  |
| A0A084EH44 | DNA ligase |  | 4.44 |  |
| B8F332 | Acetolactate synthase |  |  | 4.42 |
| A0A084ELG0 | Nicotinamide riboside transporter pnuC |  |  | 4.41 |
| A0A5C1P003 | Glycogen debranching protein GlgX | 3.03 | 3.18 |  |
| A0A084F044 | SelB translation factor | 1.62 |  | 4.38 |
| A0A143CGP5 | Malic enzyme |  | 4.37 | 1.59 |
| U4RYB7 | ABC transporter substrate-binding protein |  | 4.31 |  |
| A0A017QJ97 | Putative outer membrane protein |  |  | 4.29 |
| A0A084F0C9 | Uncharacterized protein |  | 4.27 |  |
| A0A084EGG1 | Glutathione import ATP-binding protein GsiA |  |  | 4.26 |
| A0A084EY92 | Replication-associated recombination protein A |  |  | 4.25 |
| A0A5C1P241 | CTP synthase |  |  | 4.23 |
| B8F6C1 | Zn-dependent oligopeptidase |  | 2.36 | 2.81 |
| A0A384XE99 | BAX inhibitor protein |  | 4.09 | 4.09 |
| A0A084F175 | Alanine dehydrogenase |  |  | 4.07 |
| Q3YJ04 | Predicted ATP-dependent endonuclease (Fragment) | 1.91 |  | 2.13 |
| B8F306 | DNA gyrase subunit A | 1.69 | 1.35 | 0.90 |
| A0A084EX06 | N-acetylglucosamine-6-phosphate deacetylase |  |  | 3.92 |
| A0A084EW27 | Alpha-1,4 glucan phosphorylase | 3.88 |  |  |
| A0A084EX74 | Enoyl-[acyl-carrier-protein] reductase [NADH] | 3.82 | 3.82 | 3.82 |
| A0A084F0K3 | Purine nucleoside phosphorylase DeoD-type |  | 3.77 | 3.77 |
| A0A084EAB9 | L-lactate permease |  |  | 3.77 |
| A0A1T0AE58 | 1,4-alpha-glucan branching enzyme GlgB |  | 3.75 |  |
| A0A377ILP1 | Iron-hydroxamate transporter permease subunit | 3.73 |  |  |
| A0A084EVU9 | Elongation factor P | 3.72 |  |  |
| A0A084EVE3 | Endolytic peptidoglycan transglycosylase RlpA |  | 3.64 |  |
| A0A084F161 | Neu5Ac permease |  | 3.64 |  |
| B8F7D9 | Type I restriction enzyme R Protein | 3.55 |  |  |
| A0A084EVV7 | Fumarate and nitrate reduction regulatory protein |  |  | 3.52 |
| U4SPH6 | Uncharacterized protein |  |  | 3.50 |
| A0A084F0B8 | Transaldolase |  |  | 3.48 |
| A0A5C1P089 | Phenylalanine--tRNA ligase beta subunit | 2.52 | 2.14 |  |
| B8F4B9 | ATP-dependent zinc metalloprotease FtsH |  | 3.27 |  |
| A0A084EVY7 | Ribosomal protein S12 methylthiotransferase accessory factor YcaO |  | 3.27 |  |
| A0A084EW17 | 1-deoxy-D-xylulose-5-phosphate synthase |  |  | 3.25 |
| U4RND0 | 5'-nucleotidase (Fragment) |  |  | 3.16 |
| A0A384XHY8 | Uncharacterized protein | 3.13 |  |  |
| A0A084F0B2 | CTP synthase |  |  | 3.13 |
| A0A1T0A7X4 | Integrase |  |  | 3.13 |
| A0A1T0A9V5 | Phospho-2-dehydro-3-deoxyheptonate aldolase |  |  | 3.10 |
| A0A084EYN2 | Uncharacterized protein |  |  | 3.10 |
| A0A5C1NXN7 | S24 family peptidase |  |  | 3.08 |
| A0A084EVB1 | Putative protease yhbU | 3.05 |  |  |
| A0A5C1NYJ9 | T-protein |  |  | 2.95 |
| A0A084F153 | Carbamoyl-phosphate synthase small chain |  |  | 2.94 |
| A0A143CES8 | Uncharacterized protein | 2.89 | 2.89 |  |
| A0A017QHE4 | RecBCD enzyme subunit RecB | 2.85 |  |  |
| A0A017QFR6 | ABC transporter ATP-binding protein |  |  | 2.82 |
| A0A084EY85 | Cytochrome c-type biogenesis protein CcmF |  |  | 2.79 |
| U4RR10 | Type I restriction enzyme R Protein | 2.74 |  |  |
| A0A377IKA2 | Long-chain-fatty-acid--CoA ligase |  |  | 2.68 |
| A0A143CG85 | C4-dicarboxylate ABC transporter |  | 2.62 |  |
| B8F347 | Glycosyltransferase/capsular polysaccharide phosphotransferase wcwK (Stealth protein wcwK) |  |  | 2.62 |
| A0A084ELK8 | 1-deoxy-D-xylulose 5-phosphate reductoisomerase |  |  | 2.53 |
| A0A143CEG5 | Plastocyanin |  | 2.49 |  |
| A0A084EYM1 | S1 motif domain-containing protein |  | 2.47 |  |
| A0A5C1P1S5 | Peptide chain release factor 2 |  |  | 2.47 |
| A0A084EWW3 | Fructose-1,6-bisphosphatase |  |  | 2.41 |
| A0A084EW37 | Cysteine-tRNA ligase |  |  | 2.40 |
| B8F8E3 | Type III restriction enzyme, res subunit | 2.39 |  |  |
| B8F3U5 | tRNA-cytidine(32) 2-sulfurtransferase | 2.37 |  | 2.37 |
| A0A084EVD3 | Putative 2-succinyl-6-hydroxy-2,4-cyclohexadiene-1-carboxylate synthase |  |  | 2.35 |
| A0A143CH82 | Tryptophan synthase beta chain |  |  | 2.26 |
| A0A084EVK6 | 50S ribosomal protein L3 glutamine methyltransferase | 2.23 |  |  |
| A0A084F0F8 | Anaerobic sulfatase maturase |  |  | 2.23 |
| A0A5C1P153 | S-adenosylmethionine:tRNA ribosyltransferase-isomerase |  |  | 2.22 |
| A0A084F123 | HTH-type transcriptional regulator MalT | 2.21 |  |  |
| A0A084EVB2 | Probable cytosol aminopeptidase |  |  | 2.01 |
| A0A5C1NW42 | Type II toxin-antitoxin system HipA family toxin |  |  | 2.01 |
| U4RR39 | Carbon starvation CstA family protein |  |  | 1.97 |
| A0A084EZU1 | Phage head morphogenesis protein, SPP1 gp7 family |  |  | 1.90 |
| A0A084EXV2 | Aspartate-semialdehyde dehydrogenase |  |  | 1.89 |
| A0A084EAF4 | Phosphoenolpyruvate carboxykinase (ATP) |  |  | 1.87 |
| A0A143CGT4 | Alpha-L-fucosidase |  |  | 1.80 |
| A0A1S9ZXT4 | Putative phage-like tail fiber protein |  | 1.75 |  |
| B8F782 | Glutamine synthetase |  | 1.67 |  |
| A0A084EZI9 | Phosphoserine aminotransferase |  |  | 1.67 |
| A0A084EVW8 | Putative transport protein HS327_01386 |  |  | 1.63 |
| A0A084EVG7 | Putative ABC transporter ATP-binding protein |  |  | 1.62 |
| A0A084F0F5 | DNA recombination protein RmuC-like protein |  |  | 1.49 |
| A0A143CGN9 | Sodium/panthothenate symporter |  | 1.47 | 1.47 |
| U4S473 | DNA translocase ftsK | 1.39 |  |  |
| A0A084ELK5 | GTPase Der | 1.39 |  |  |
| A0A145QX48 | Sulfatase domain-containing protein |  | 1.39 |  |
| U4RN40 | Aspartate--tRNA ligase |  | 1.36 |  |
| A0A1T0A8I4 | Restriction endonuclease subunit S |  |  | 1.36 |
| A0A084F0P4 | 5-methyltetrahydropteroyltriglutamate--homocysteine methyltransferase |  | 1.32 |  |
| A0A145QRD2 | Uncharacterized protein | 1.07 | 1.07 | 1.07 |
| A0A084EZG9 | Soluble lytic murein transglycosylase |  |  | 1.01 |
| A0A084EEW0 | Serotype-specific antigen 1 |  |  | 0.86 |
| A0A145QVP5 | Type I restriction enzyme R Protein | 0.78 |  |  |
| A0A084EH31 | Uncharacterized protein | 0.78 |  |  |
| A0A084EX80 | Acriflavine resistance protein B | 0.68 |  |  |

^a^ The percentage of the protein sequence covered (coverage) by identified peptides in L-1 negative bands.
