# Supplementary figures and images for: Cleavage of E-cadherin by porcine respiratory bacterial pathogens facilitates airway epithelial barrier disruption and bacterial paracellular transmigration
Source: Virulence. 2021 Sep 5;12(1):2296–313. doi: 10.1080/21505594.2021.1966996 (PMC8425755; doi:10.1080/21505594.2021.1966996)

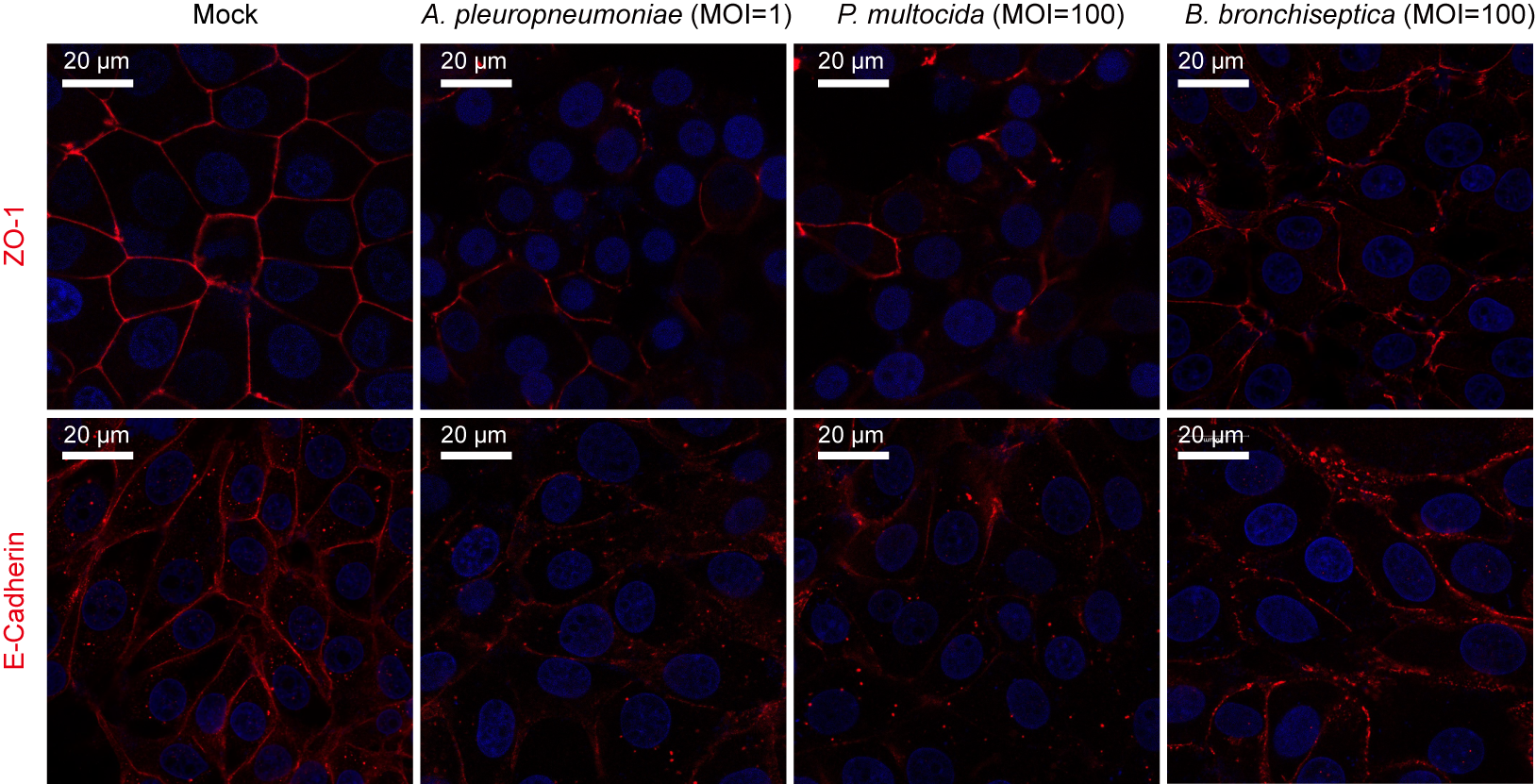

Supplement: Supplemental Material [file KVIR_A_1966996_SM6607.zip › suppl/Fig S1.tif]

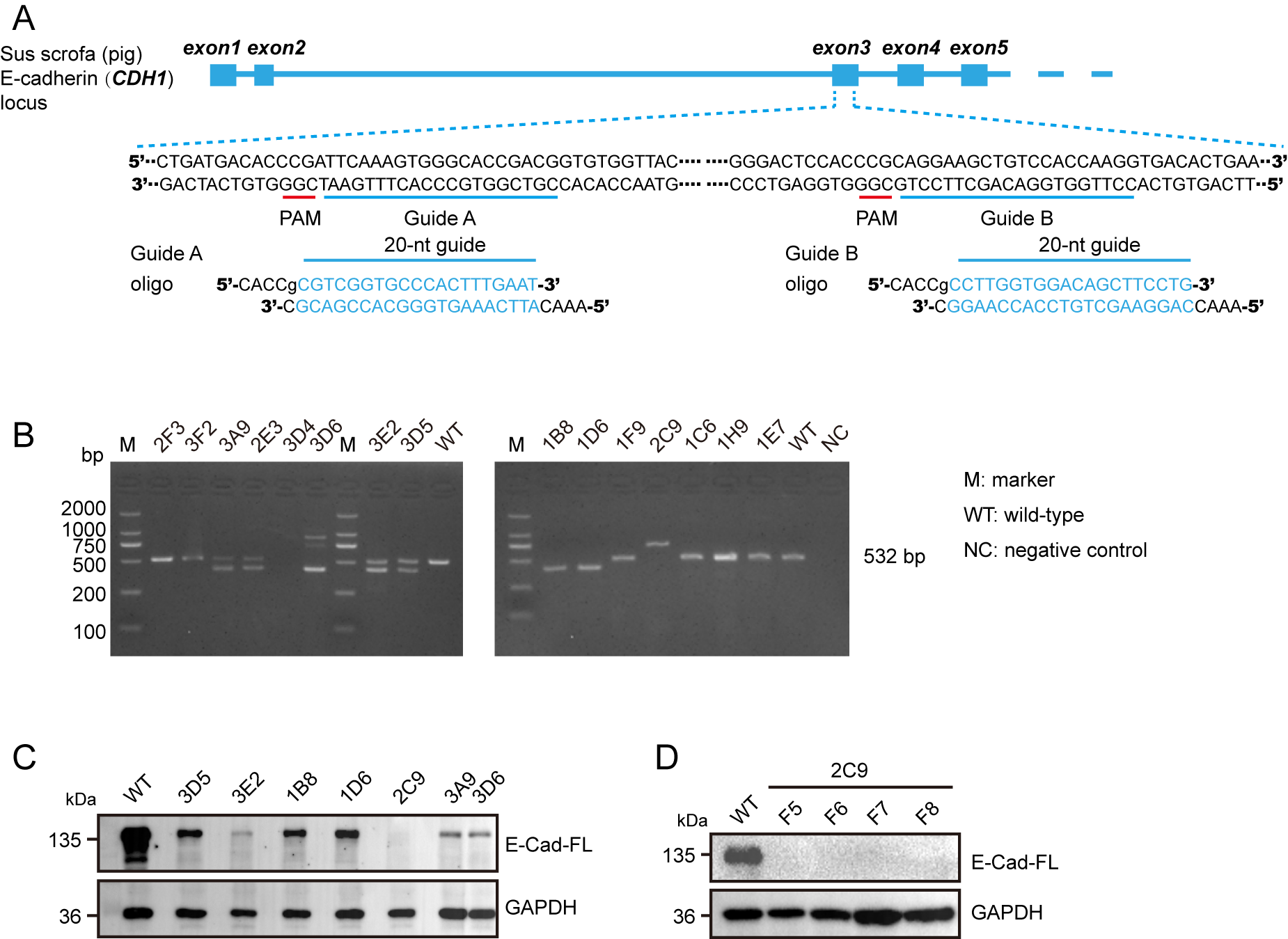

Supplement: Supplemental Material [file KVIR_A_1966996_SM6607.zip › suppl/Fig S2.tif]

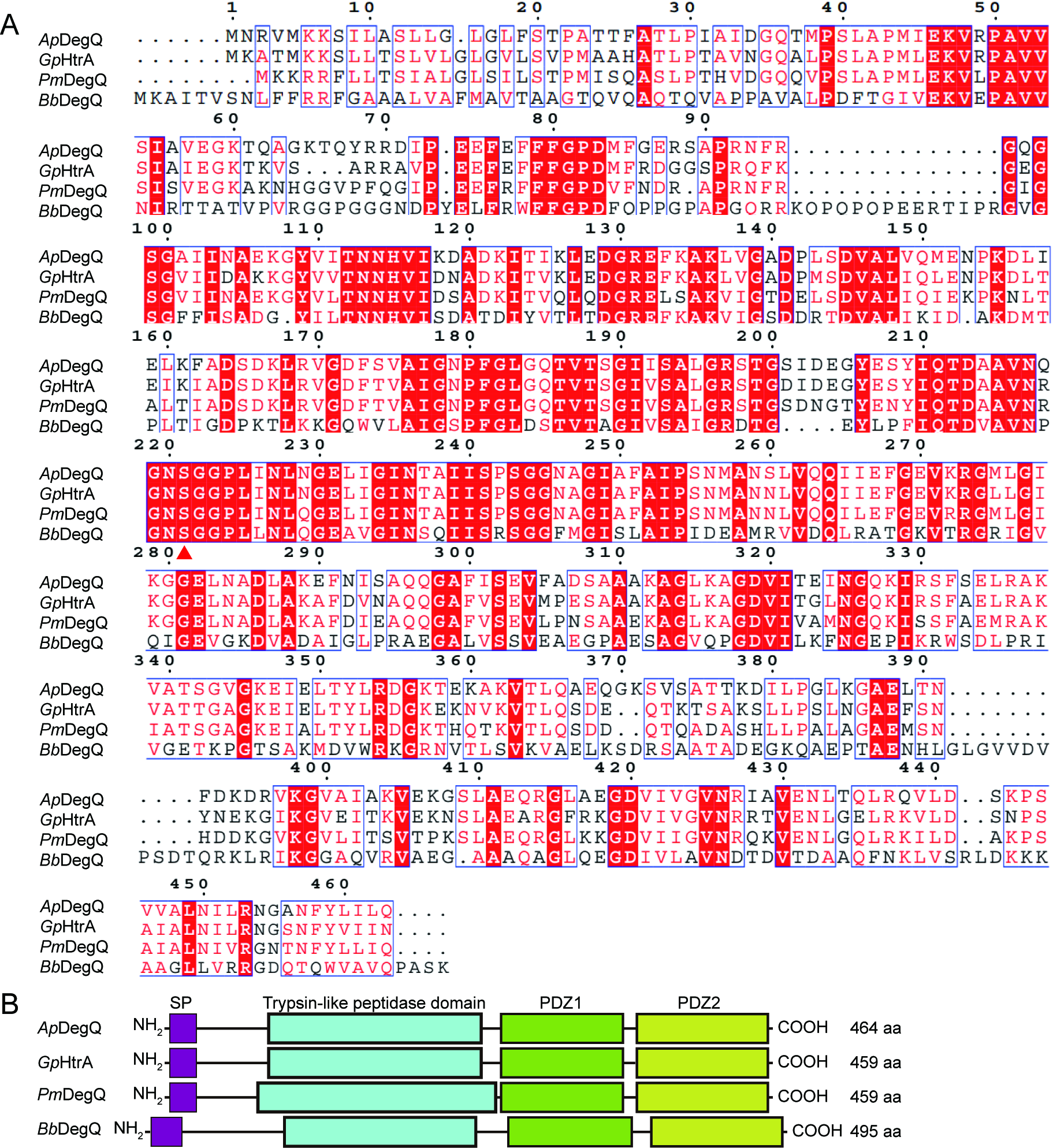

Supplement: Supplemental Material [file KVIR_A_1966996_SM6607.zip › suppl/Fig S3.tif]

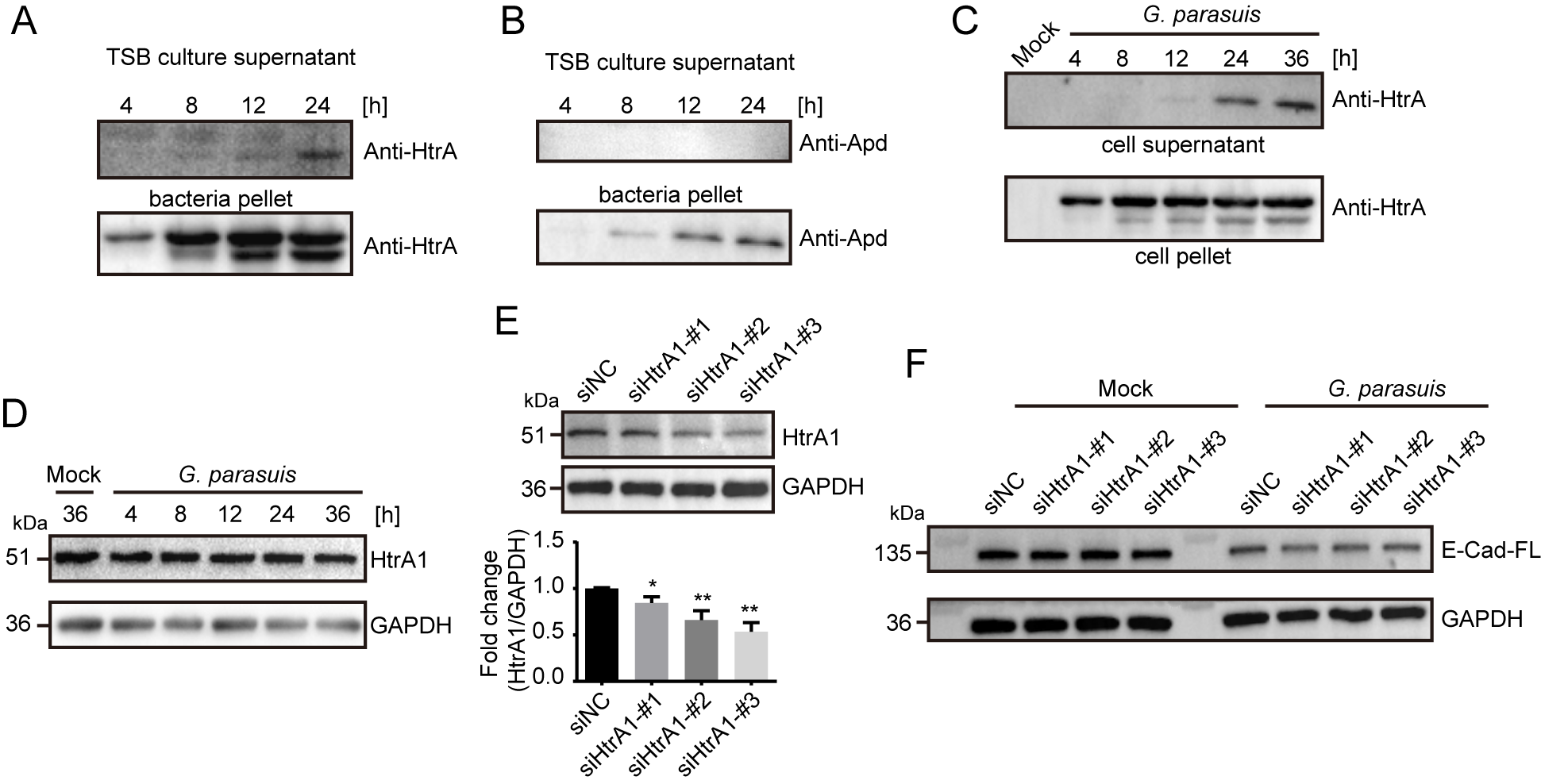

Supplement: Supplemental Material [file KVIR_A_1966996_SM6607.zip › suppl/Fig S4.tif]

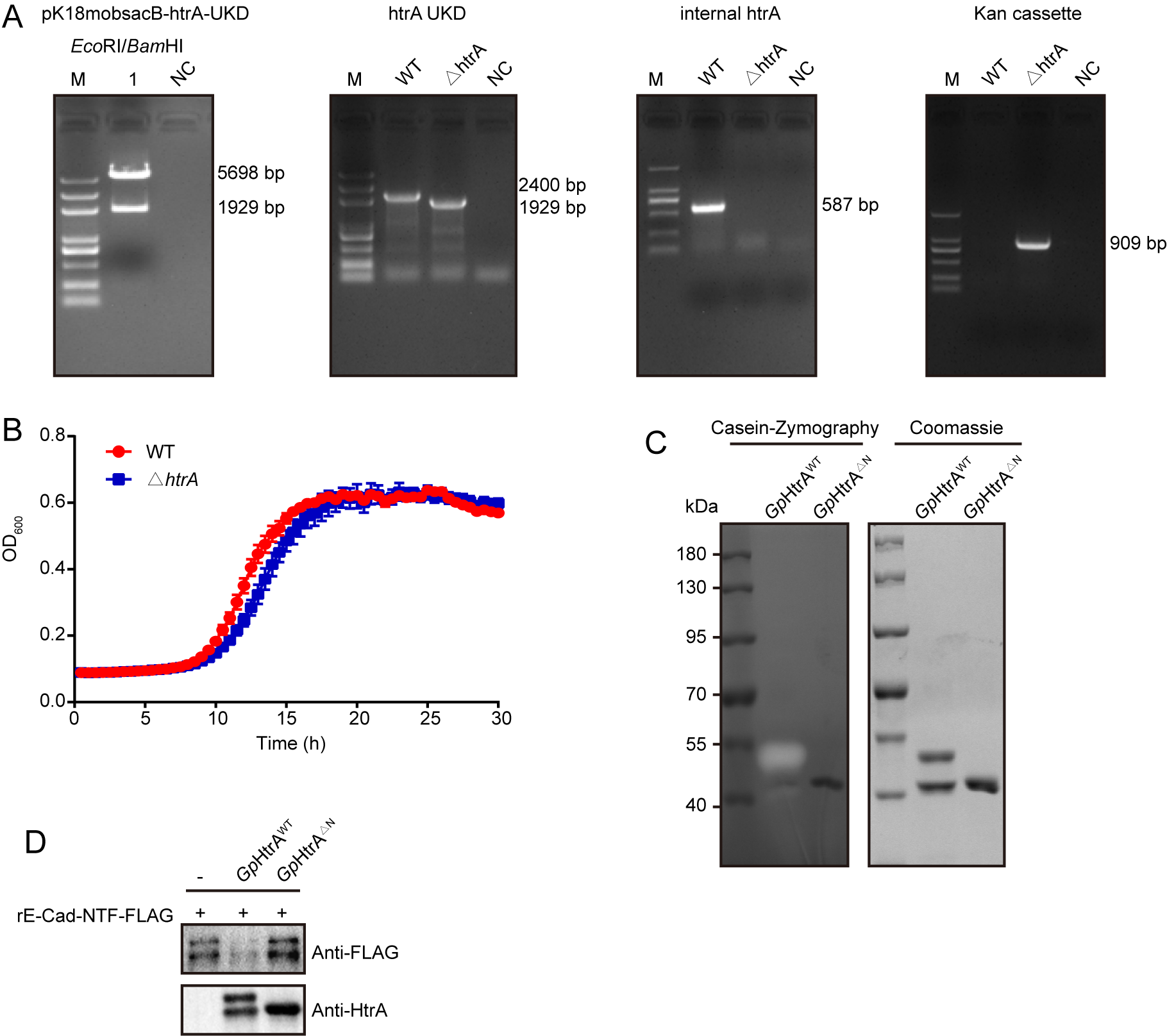

Supplement: Supplemental Material [file KVIR_A_1966996_SM6607.zip › suppl/Fig S5.tif]

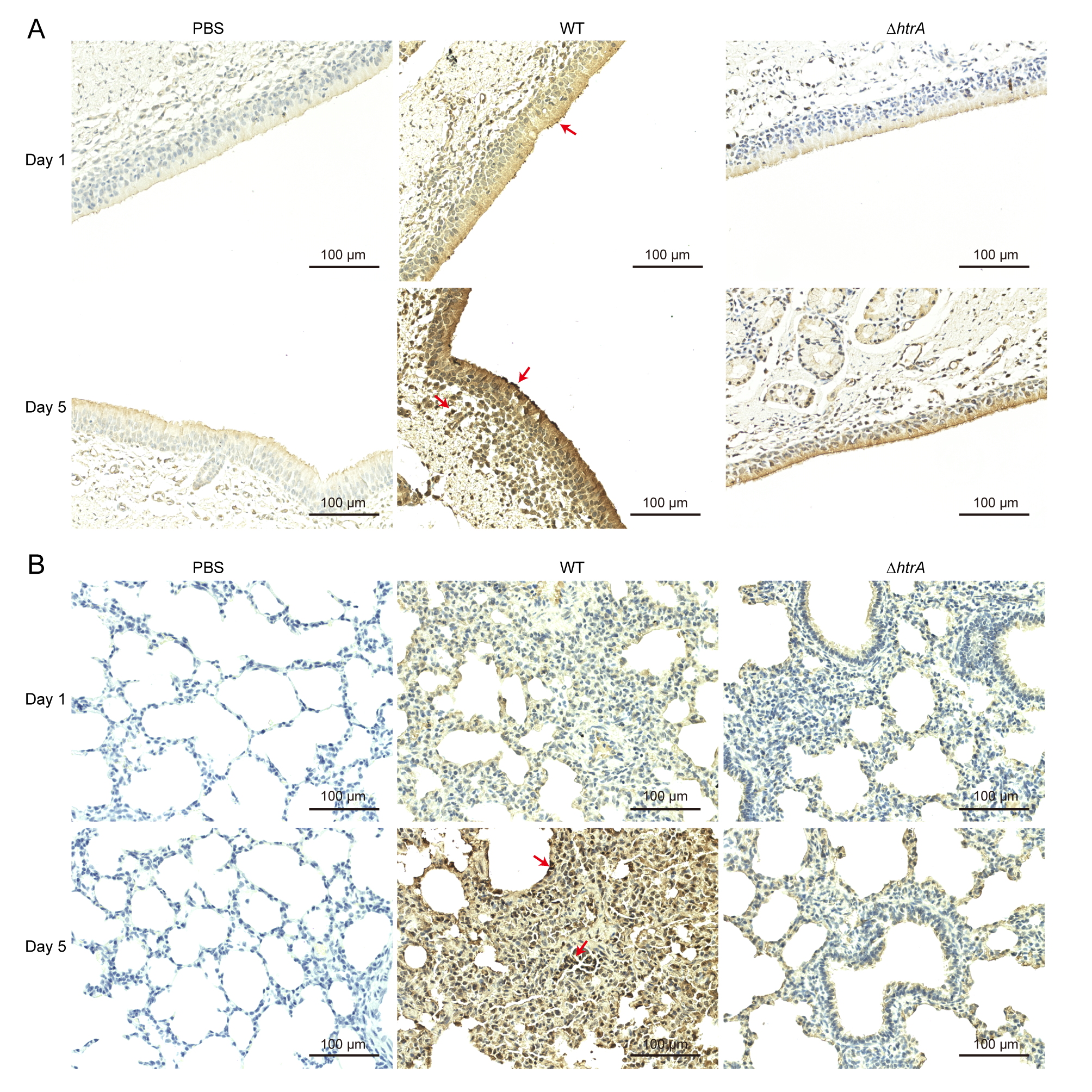

Supplement: Supplemental Material [file KVIR_A_1966996_SM6607.zip › suppl/Fig S6.tif]
